# Genetic basis of cardiomyopathy and the genotypes involved in prognosis and left ventricular reverse remodeling

**DOI:** 10.1038/s41598-018-20114-9

**Published:** 2018-01-31

**Authors:** Takashige Tobita, Seitaro Nomura, Takanori Fujita, Hiroyuki Morita, Yoshihiro Asano, Kenji Onoue, Masamichi Ito, Yasushi Imai, Atsushi Suzuki, Toshiyuki Ko, Masahiro Satoh, Kanna Fujita, Atsuhiko T Naito, Yoshiyuki Furutani, Haruhiro Toko, Mutsuo Harada, Eisuke Amiya, Masaru Hatano, Eiki Takimoto, Tsuyoshi Shiga, Toshio Nakanishi, Yasushi Sakata, Minoru Ono, Yoshihiko Saito, Seiji Takashima, Nobuhisa Hagiwara, Hiroyuki Aburatani, Issei Komuro

**Affiliations:** 1 Department of Cardiology, Tokyo Women’s Medical University, Tokyo, Japan; 2 Research Fellow of Japan Society for the Promotion of Science, Tokyo, Japan; 3 Genome Science Division, Research Center for Advanced Science and Technology, The University of Tokyo, Tokyo, Japan; 4 Department of Cardiovascular Medicine, Graduate School of Medicine, The University of Tokyo, Tokyo, Japan; 5 Department of Cardiovascular Medicine, Osaka University Graduate School of Medicine, Osaka, Japan; 6 First Department of Internal Medicine, Nara Medical University, Kashihara, Japan; 7 Division of Cardiovascular Medicine, Jichi Medical University, Shimotsuke, Japan; 8 Department of Cardiovascular Medicine, Chiba University Graduate School of Medicine, Chiba, Japan; 9 Department of Pediatric Cardiology, Tokyo Women’s Medical University, Tokyo, Japan; 10 Department of Cardiovascular Surgery, Graduate School of Medicine, The University of Tokyo, Tokyo, Japan; 11 Department of Medical Biochemistry, Osaka University Graduate School of Medicine, Osaka, Japan

## Abstract

Dilated cardiomyopathy (DCM) and hypertrophic cardiomyopathy (HCM) are genetically and phenotypically heterogeneous. Cardiac function is improved after treatment in some cardiomyopathy patients, but little is known about genetic predictors of long-term outcomes and myocardial recovery following medical treatment. To elucidate the genetic basis of cardiomyopathy in Japan and the genotypes involved in prognosis and left ventricular reverse remodeling (LVRR), we performed targeted sequencing on 120 DCM (70 sporadic and 50 familial) and 52 HCM (15 sporadic and 37 familial) patients and integrated their genotypes with clinical phenotypes. Among the 120 DCM patients, 20 (16.7%) had *TTN* truncating variants and 13 (10.8%) had *LMNA* variants. *TTN* truncating variants were the major cause of sporadic DCM (21.4% of sporadic cases) as with Caucasians, whereas *LMNA* variants, which include a novel recurrent *LMNA* E115M variant, were the most frequent in familial DCM (24.0% of familial cases) unlike Caucasians. Of the 52 HCM patients, *MYH7* and *MYBPC3* variants were the most common (12 (23.1%) had *MYH7* variants and 11 (21.2%) had *MYBPC3* variants) as with Caucasians. DCM patients harboring *TTN* truncating variants had better prognosis than those with *LMNA* variants. Most patients with *TTN* truncating variants achieved LVRR, unlike most patients with *LMNA* variants.

## Introduction

Dilated cardiomyopathy (DCM) and hypertrophic cardiomyopathy (HCM) are genetic disorders that cause heart failure and life-threatening arrhythmia, eventually requiring heart transplantation or cardiac device implantation^1^. These cardiomyopathies have prevalence rates of approximately 0.004% and 0.2%, respectively, with familial cases accounting for 20–50% of all cases^2–4^. Currently, more than 50 genes have been reported to be associated with these cardiomyopathies^5^, with some ethnic-specific founder mutations^6,7^. Considering that racial differences could affect mutational profiles, the genetic basis of these disorders in Japanese patients might be different from that in Caucasian patients.

Since patients with cardiomyopathies show diverse clinical phenotypes, the precise prediction of prognosis is difficult in the clinical setting^8^. There have been some reports showing the particular phenotypes corresponding to specific genotypes. For example, *LMNA* mutations in DCM patients have been reported to be linked to a high incidence of sudden cardiac death^9,10^. Identification of the genotypes involved in prognosis and treatment response would contribute to risk stratification and accurate treatment decisions.

Left ventricular (LV) reverse remodeling (LVRR) is known to occur under medical treatment in approximately 40% of DCM patients^11,12^. DCM patients who achieved LVRR have better prognosis^11^; however, the specific genotypes involved in LVRR have been elusive. In this study, we explore the genetic basis and novel genotype–phenotype associations in Japanese patients with cardiomyopathies and elucidate the genotypes involved in clinical prognosis and LVRR.

## Results

### Study population

We analyzed a Japanese cardiomyopathy cohort consisting of 120 unrelated DCM patients and 52 unrelated HCM patients. Their baseline characteristics and clinical information during follow-up are summarized in Table 1a and b. Of the 120 DCM patients, 50 had familial DCM, 82.5% were men, and the mean age at diagnosis was 39.1 ± 13.9 years. Twenty-two patients underwent heart transplantation, and 11 died. Of the 52 HCM patients, 37 had familial HCM, 61.5% were men, and the mean age at diagnosis was 31.4 ± 17.1 years. Six patients underwent heart transplantation, and 1 patient died. DCM and HCM patients generally underwent endomyocardial biopsy, and the diagnosis was determined as accurately as possible.

**Table 1:** Clinical features at baseline and clinical information during follow-up in DCM patients.

	DCM (n = 120)
(**a**)	
Age at diagnosis (years)	39.1 ± 13.9
Male	99 (82.5%)
Familial	50 (41.7%)
Familial history of sudden death	23 (19.2%)
NYHA functional class ≥3	65/116 (56.0%)
B-type natriuretic peptide (pg/ml)	325 (109–1037)
Cardiac catheterization	117 (97.5%)
Endomyocardial biopsy (n = 96)	
Inflammation	17/96 (17.7%)
Fibrosis	86/96 (89.6%)
Echocardiography (n = 113)	
LVEF (%)	29.9 ± 12.4
LVEDD (mm)	66.2 ± 11.4
LVESD (mm)	57.9 ± 13.8
Interventricular septum (mm)	7.8 ± 2.2
Posterior wall (mm)	7.9 ± 2.4
LV mass (g)	223.3 ± 89.5
LAD (mm)	44.4 ± 9.6
Restrictive mitral pattern (%)	30/74 (40.5%)
E/e’	14.4 ± 10.1
Mitral regurgitation ≥ moderate	37/108 (34.3%)
Left ventricular reverse remodeling	20/45 (44.4%)
Cardiopulmonary exercise testing (n = 42)	
Rest exercise heart rate (beats/min)	79 ± 15
Peak exercise heart rate (beats/min)	126 ± 29
Rest exercise systolic blood pressure (mmHg)	94 ± 18
Peak exercise systolic blood pressure (mmHg)	130 ± 31
Peak VO_2_ (mL/kg/min)	15.4 ± 6.6
Follow-up data	
Amiodarone	57 (47.5%)
Pacemaker implantation	2 (1.7%)
ICD implantation	14 (11.7%)
CRT-D implantation	36 (30.0%)
ICD or CRT-D implantation	50 (41.7%)
Any device	54 (45.0%)
AF	36 (30.0%)
Non-sustained VT	59 (49.2%)
Sustained VT	30 (25.0%)
VF, CPR	17 (14.2%)
Heart transplantation	22 (18.3%)
Mortality	11 (9.2%)
Heart transplantation or Mortality	33 (27.5%)
Mean follow-up duration (years)	8.7 ± 8.3
**(b)**	
	**HCM (n = 52)**
Age at diagnosis (years)	31.4 ± 17.1
Male	32 (61.5%)
Familial	37 (71.2%)
Familial history of sudden death	18 (34.6%)
NYHA functional class ≥3	20 (38.5%)
B-type natriuretic peptide (pg/ml)	339 (110–832)
Cardiac catheterization	45 (86.5%)
Endomyocardial biopsy (n = 38)	
Inflammation	3/38 (7.9%)
Fibrosis	37/38 (97.4%)
Echocardiography (n = 48)	
LVEF (%)	50.9 ± 20.6
LVEDD (mm)	52.2 ± 13.5
LVESD (mm)	38.9 ± 17.8
Interventricular septum (mm)	13.0 ± 4.4
Posterior wall (mm)	9.4 ± 2.8
LV mass (g)	227.9 ± 103.6
LAD (mm)	44.1 ± 10.6
Restrictive mitral pattern (%)	12/41 (29.3%)
E/e’	13.7 ± 7.9
Mitral regurgitation ≥ moderate	7/47 (14.9%)
Maximum wall thickness	13.7 ± 4.5
Peak LVOT gradient ≥30 mmHg	5/48 (10.4%)
Cardiopulmonary exercise testing (n = 18)	
Rest exercise heart rate (beats/min)	71 ± 9
Peak exercise heart rate (beats/min)	104 ± 29
Rest exercise systolic blood pressure (mmHg)	94 ± 18
Peak exercise systolic blood pressure (mmHg)	123 ± 31
Peak VO_2_ (mL/kg/min)	11.8 ± 2.9
Follow-up data	
Amiodarone	28 (53.8%)
Pacemaker implantation	0
ICD implantation	10 (19.2%)
CRT-D implantation	17 (32.7%)
ICD or CRT-D implantation	27 (51.9%)
Any device	28 (53.8%)
AF	22 (42.3%)
Non-sustained VT	20 (38.5%)
Sustained VT	9 (17.3%)
VF, CPR	9 (17.3%)
End-stage HCM (LVEF <50%)	31 (59.6%)
Heart transplantation	6 (11.5%)
Mortality	1 (1.9%)
Heart transplantation or Mortality	7 (13.5%)
Mean follow-up duration (years)	17.1 ± 12.1

Values are n (%), the mean ± SD, or median (interquartile). HCM, hypertrophic cardiomyopathy; NYHA, New York Heart Association; LV, left ventricular; EF, ejection fraction; EDD, end-diastolic diameter; ESD, end-systolic diameter; LAD, left atrial dimension; LVOT, left ventricular outflow tract; ICD, implantable cardioverter defibrillator; VF, ventricular fibrillation; CRTD, cardiac resynchronization therapy defibrillator; AF, atrial fibrillation; VT, ventricular tachycardia; CPR, cardiopulmonary resuscitation.

### Sequencing summary and filtering variants

**Figure 1:**
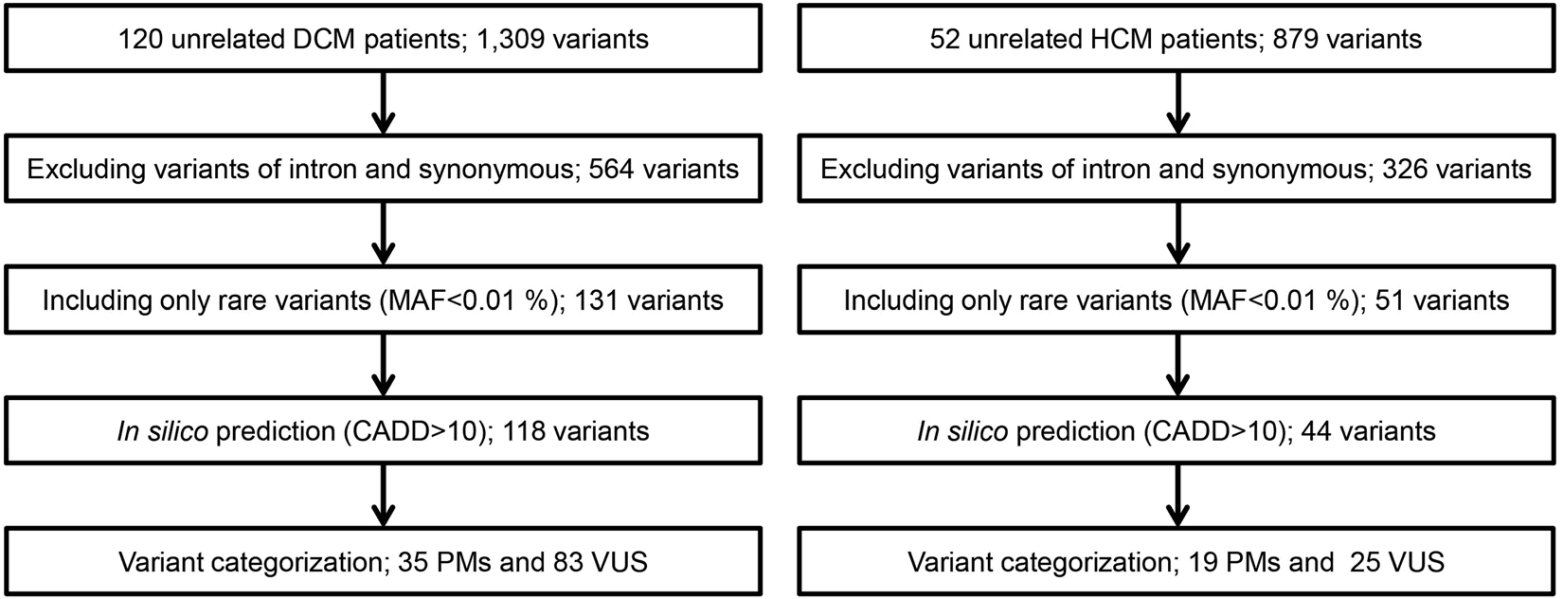
Flowchart summarizing the variant categorization. The number of variants is described in each category. DCM, dilated cardiomyopathy; HCM, hypertrophic cardiomyopathy; PM, pathogenic mutation; VUS, variant of uncertain significance; MAF, minor allele frequency; CADD, combined annotation-dependent depletion.

From the 172 genomic DNA samples, we sequenced 95 genes, including the exonic and splicing regions (Supplementary Table 1). The median read depth in the target region was 380×, and 98.6% of the target regions had a read depth of over 20×. Variant filtering was conducted as shown in Fig. 1. We identified 1,309 variants among DCM patients, 564 of which were in exonic nonsynonymous or splice site regions. After excluding variants with minor allele frequencies greater than 0.01%, we identified 131 variants, among which 118 variants were predicted to be deleterious based on combined annotation-dependent depletion (CADD) scores. Finally, 35 pathogenic mutations (PMs) and 83 variants of uncertain significance (VUSs) were identified in the DCM cohort (Supplementary Table 2). In the HCM patients, we identified 879 variants, 326 of which were in exonic nonsynonymous or splice site regions. After excluding variants with minor allele frequencies greater than 0.01%, we identified 51 variants, among which 44 variants were predicted to be deleterious based on CADD scores. Finally, 19 PMs and 25 VUSs were identified in the HCM cohort (Supplementary Table 3).

### Genetic basis of DCM and HCM

**Figure 2:**
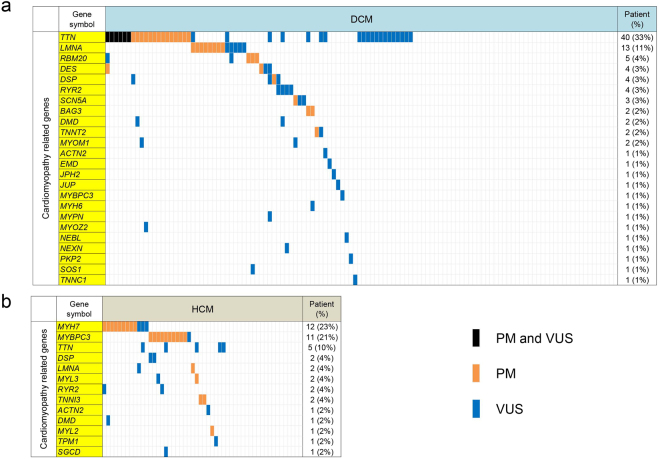
Genetic profiles of cardiomyopathies. Genetic profiles of DCM (**a**) and HCM (**b**) are represented. Only genes closely linked to cardiomyopathy (yellow) are shown. Colored cells represent the presence of PM (orange), VUS (blue), or PM and VUS (black). DCM, dilated cardiomyopathy; HCM, hypertrophic cardiomyopathy; PM, pathogenic mutation; VUS, variant of uncertain significance.

The genetic basis of DCM and HCM are summarized in Fig. 2 and Supplementary Fig. 1. In our DCM cohort, 78 patients (65.0%) had variants. *TTN* variants were the most frequent variants, and 54 rare *TTN* variants including 20 PMs were observed in 40 patients. All of these 20 PMs were considered to cause truncation. These 20 PMs had not been reported previously, and 17 of these 20 PMs were located in A-band regions. Ten rare *LMNA* variants were the second-most frequent and were found in 13 patients (7 PMs in 8 patients). Five of these 7 PMs were truncating variants. In addition, 2 PMs were found in *BAG3* and *RBM20*. In our DCM cohort, most variants were private and only 4 of the 118 variants were detected in unrelated patients; in particular, a novel VUS in *LMNA* (p.E115M) was shared by 3 unrelated patients (Supplementary Table 4).

Among the 52 HCM patients, 35 patients (67.3%) had variants. *MYH7* and *MYBPC3* variants were the most common, and 23 HCM patients had variants in these genes. Ten rare *MYH7* variants were found in 12 patients (8 PMs in 9 patients). Eight rare *MYBPC3* variants were found in 11 patients (7 PMs in 10 patients). *TTN* truncating variants were not observed in HCM patients. In the HCM cohort, 6 variants were detected in unrelated patients; in particular, a novel VUS in *MYH7* (p.E504G) was shared by 2 unrelated patients (Supplementary Table 4).

### Multiple rare variants and variant classes in cardiomyopathy patients

In our DCM cohort, 37 patients (30.8%) had PMs and only 1 patient had 2 PMs. Thirty-three patients (27.5%) had multiple variants, and 6.7% of patients had 3 or more variants (Supplementary Table 5). In the HCM cohort, 24 patients (46.2%) had PMs, and none had multiple PMs. Fourteen patients (26.9%) had multiple variants, and 3.8% of patients had three variants (Supplementary Table 5). We then evaluated the association between variant classes and phenotypes. DCM patients with any variants were likely to have a family history, whereas this relationship was not observed in HCM patients (Supplementary Tables 6 and 7). HCM patients with PMs developed AF and required device implantation during follow-up (Supplementary Table 7). Both in DCM and HCM, the clinical outcomes of life-threatening arrhythmia, heart transplantation, and mortality were not associated with variant classes (Supplementary Tables 6 and 7). Additionally, there were no significant differences in the primary endpoint according to variant classes or number of variants in DCM patients (Supplementary Figs 2 and 3).

### Genotype–phenotype associations in DCM patients

**Figure 3:**
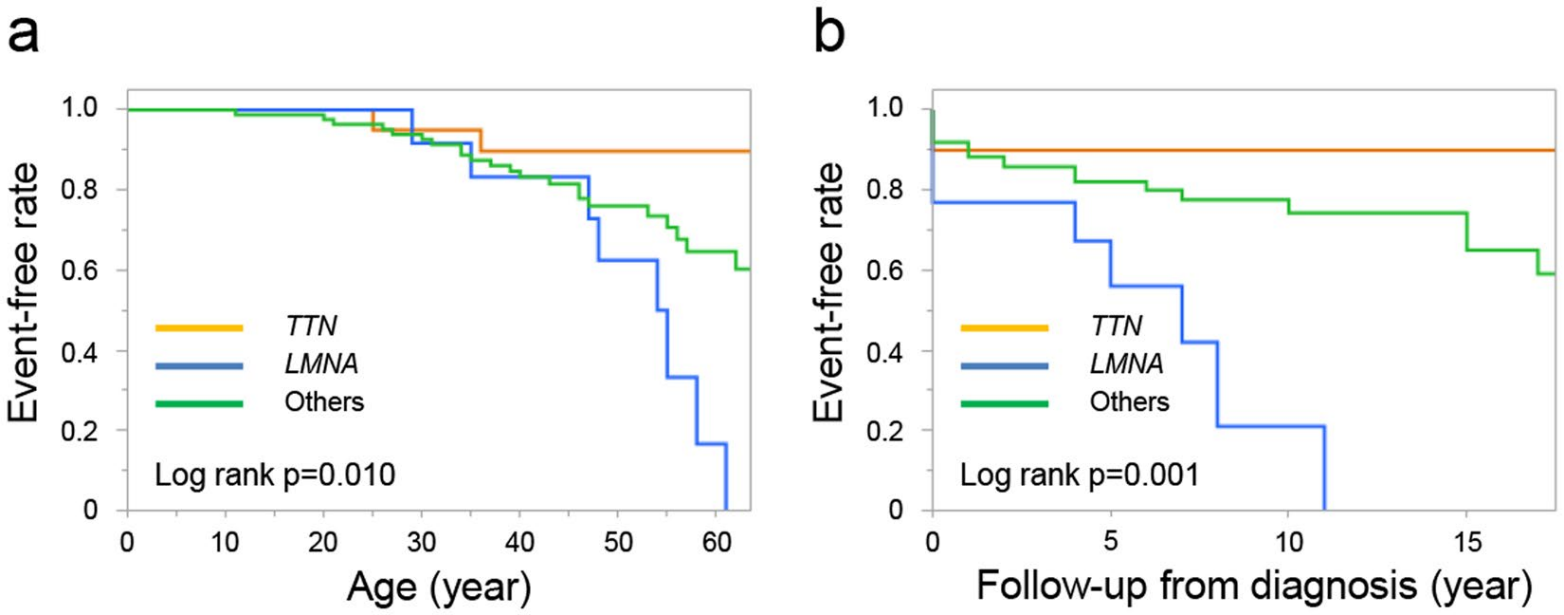
Survival free of life-threatening arrhythmia in DCM patients with *TTN* truncating variants (n = 20), DCM patients with *LMNA* variant (n = 13), and other DCM patients (n = 87). Kaplan–Meier curves illustrating survival free of life-threatening arrhythmia throughout lifespan (**a**) and during follow-up (**b**). Probability values were calculated using log-rank tests. DCM, dilated cardiomyopathy.

**Figure 4:**
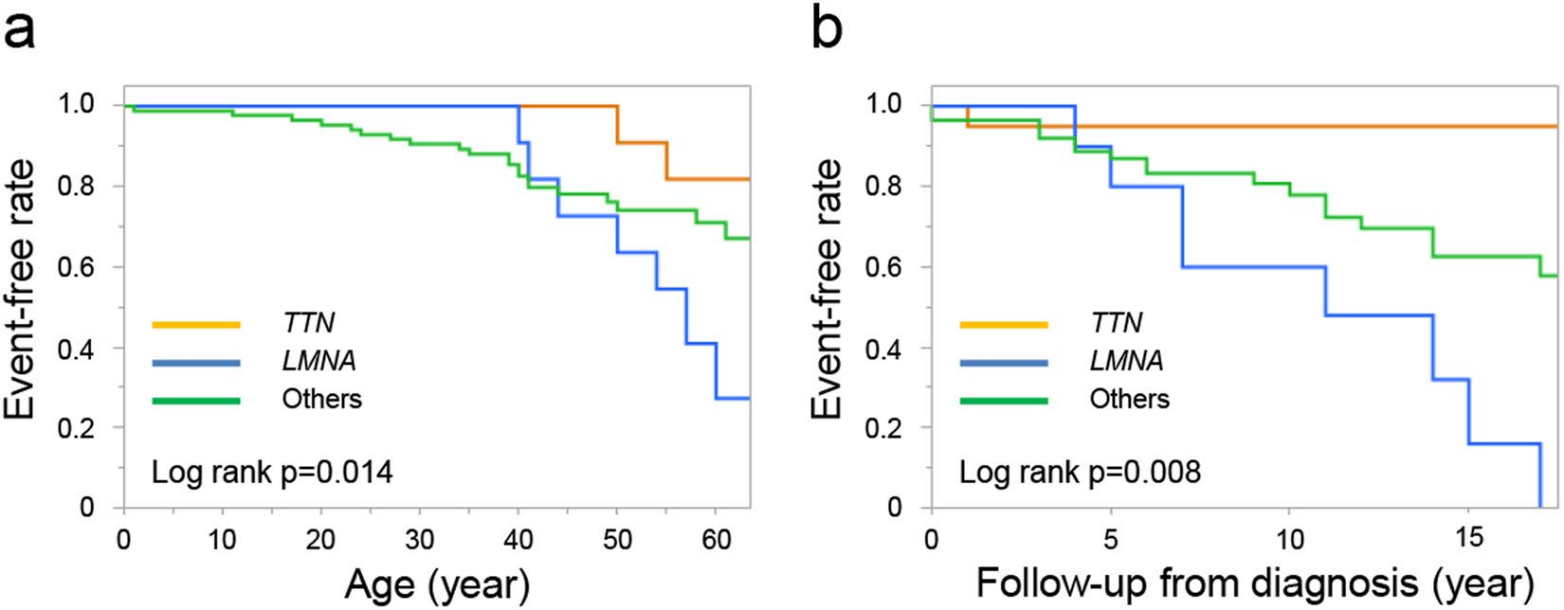
Heart transplant- or death-free survival in DCM patients with *TTN* truncating variants (n = 20), DCM patients with *LMNA* variants (n = 13), and other DCM patients (n = 87). Kaplan–Meier curves illustrating heart transplant- or death-free survival throughout lifespan (**a**) and during follow-up (**b**). Probability values were calculated using log-rank tests. DCM, dilated cardiomyopathy.

We then divided DCM patients into three groups consisting of those with *TTN* truncation, those with *LMNA* variants, and others, to investigate genotype–phenotype associations (Table 2) for common clinical characteristics and outcomes. In the *TTN* truncation group, 5 patients (25.0%) had familial history, and all probands were men. Family history of sudden death was found in only 1 family. Additionally, 1 patient underwent heart transplantation, and 1 patient died from cardiac-related causes, indicating that only 2 patients reached the primary endpoint (heart transplantation and death). In the *LMNA* variant group, 11 patients (84.6%) were male, and most cases (92.3%) had familial cardiomyopathy. Family history of sudden cardiac death was found in 9 families (69.2%). Seven patients (53.8%) had sustained ventricular tachycardia. The frequency of life-threatening arrhythmias was higher in this group (Fig. 3). Five patients (38.5%) underwent heart transplantation, and 3 patients (23.1%) died during follow-up. In total, 8 patients reached the primary endpoint. Among these 8 patients, 5 had PMs and 3 patients had VUSs in the *LMNA* gene. Outcomes were better in patients with *TTN* truncating variants, whereas patients with *LMNA* variants had worse outcomes and life-threatening arrhythmic events (Figs 3 and 4).

**Table 2:** Genotype–phenotype associations in DCM patients.

	*TTN* (n = 20)	*LMNA* (n = 13)	Others (n = 87)	*p* value, *TTN* vs *LMNA*	*p* value, *TTN* vs Others	*p* value, *LMNA* vs Others
Age at diagnosis (years)	44.2 ± 11.7	41.4 ± 11.1	37.7 ± 14.6	0.519	0.062	0.370
Male	20 (100%)	11 (84.6%)	68 (78.2%)	0.148	0.021	0.731
Familial^*,#^	5 (25.0%)	12 (92.3%)	33 (37.9%)	<0.001	0.313	<0.001
Familial history of sudden death^*,#^	1 (5.0%)	9 (69.2%)	13 (14.9%)	<0.001	0.460	<0.001
NYHA functional class ≥3^†^	5/19 (26.3%)	7/12 (58.3%)	53/85 (62.4%)	0.130	0.005	0.763
B-type natriuretic peptide (pg/ml)	261 (87–870)	278 (56–549)	358 (112–1378)	0.984	0.249	0.391
Cardiac catheterization	19 (95.0%)	13 (100%)	85 (97.7%)	1.000	0.343	1.000
Endomyocardial biopsy (n = 96)						
Inflammation	1/15 (6.7%)	3/10 (30.0%)	13/71 (18.3%)	0.267	0.447	0.405
Fibrosis	12/15 (80%)	10/10 (100%)	64/71 (90.1%)	0.250	0.369	0.588
Echocardiography (n = 113)						
LVEF (%)	25.7 ± 8.9	34.3 ± 11.8	30.2 ± 13.1	0.059	0.220	0.196
LVEDD (mm)	65.8 ± 8.9	60.3 ± 8.4	67.1 ± 12.1	0.088	0.791	0.037
LVESD (mm)	58.9 ± 10.7	49.8 ± 10.7	58.8 ± 14.6	0.023	0.865	0.022
IVST (mm)	7.4 ± 2.0	7.5 ± 2.8	8.0 ± 2.1	0.750	0.403	0.350
PWT (mm)	8.0 ± 2.1	7.5 ± 2.7	7.9 ± 2.5	0.495	0.861	0.571
LV mass (g)	214.5 ± 69.1	176.2 ± 69.4	231.9 ± 94.6	0.101	0.599	0.053
LAD (mm)	43.4 ± 7.7	44.1 ± 13.2	44.7 ± 9.6	0.914	0.639	0.631
Restrictive mitral pattern (%)	4/11 (36.4%)	1/7 (14.3%)	25/56 (44.6%)	0.596	0.745	0.224
E/e’	12.4 ± 10.2	14.6 ± 12.1	14.9 ± 10.0	1.000	0.149	0.861
Mitral regurgitation ≥ moderate	7/18 (38.9%)	3/11 (27.3%)	27/79 (34.2%)	0.694	0.786	0.746
Left ventricular reverse remodeling*	9/11 (81.8%)	0/7 (0%)	11/27 (40.7%)	0.002	0.033	0.069
Cardiopulmonary exercise testing (n = 42)						
Rest exercise heart rate (beats/min)	78 ± 11	72 ± 13.5	80 ± 16	1.000	0.584	0.441
Peak exercise heart rate (beats/min)	135 ± 24	106 ± 35	125 ± 29	0.179	0.497	0.180
Rest exercise systolic blood pressure (mmHg)	96 ± 15	92 ± 14	94 ± 19	0.831	0.258	1.000
Peak exercise systolic blood pressure (mmHg)	140 ± 23	123 ± 38	127 ± 33	0.479	0.156	0.702
Peak VO_2_ (mL/kg/min)	17.6 ± 4.7	15.5 ± 10.4	14.6 ± 6.6	0.321	0.045	0.977
Follow-up data						
Amiodarone^#^	11 (55.0%)	11 (84.6%)	35 (40.2%)	0.132	0.317	0.005
Pacemaker implantation	0	0	2 (2.3%)	NA	1.000	1.000
ICD implantation	3 (15.0%)	1 (7.7%)	10 (11.5%)	1.000	0.706	1.000
CRT-D implantation^#^	9 (45.0%)	8 (61.5%)	19 (21.8%)	0.481	0.048	0.006
ICD or CRT-D implantation	12 (60.0%)	9 (69.2%)	29 (33.3%)	0.719	0.040	0.028
Any device	12 (60.0%)	9 (69.2%)	33 (37.9%)	0.719	0.083	0.040
AF	9 (45.0%)	7 (53.8%)	20 (23.0%)	0.728	0.055	0.039
Non-sustained VT^#^	11 (55.0%)	12 (92.3%)	36 (41.4%)	0.050	0.322	<0.001
Sustained VT	4 (20.0%)	7 (53.8%)	19 (21.8%)	0.065	1.000	0.036
VF, CPR	2 (10.0%)	2 (15.4%)	13 (14.9%)	1.000	0.732	1.000
Heart transplantation	1 (5.0%)	5 (38.5%)	16 (18.4%)	0.025	0.187	0.139
Mortality	1 (5.0%)	3 (23.1%)	7 (8.0%)	0.276	1.000	0.120
Heart transplantation or Mortality*	2 (10.0%)	8 (61.5%)	23 (26.4%)	0.005	0.150	0.021
Mean follow-up duration (years)	8.6 ± 8.0	8.3 ± 5.0	8.8 ± 8.8	0.711	0.752	0.531

Values are n (%), the mean ± SD, or median (interquartile). Superscript letters represent significant differences compared with other groups (^*^TTN group versus *LMNA* group; ^†^*TTN* group versus others group; ^#^*LMNA* group versus others group). DCM, dilated cardiomyopathy; NYHA, New York Heart Association; LV, left ventricular; EF, ejection fraction; EDD, end-diastolic diameter; ESD, end-systolic diameter; LAD, left atrial dimension; ICD, implantable cardioverter defibrillator; VF, ventricular fibrillation; CRTD, cardiac resynchronization therapy defibrillator; AF, atrial fibrillation; VT, ventricular tachycardia; CPR, cardiopulmonary resuscitation; NA, not applicable.

**Figure 5:**
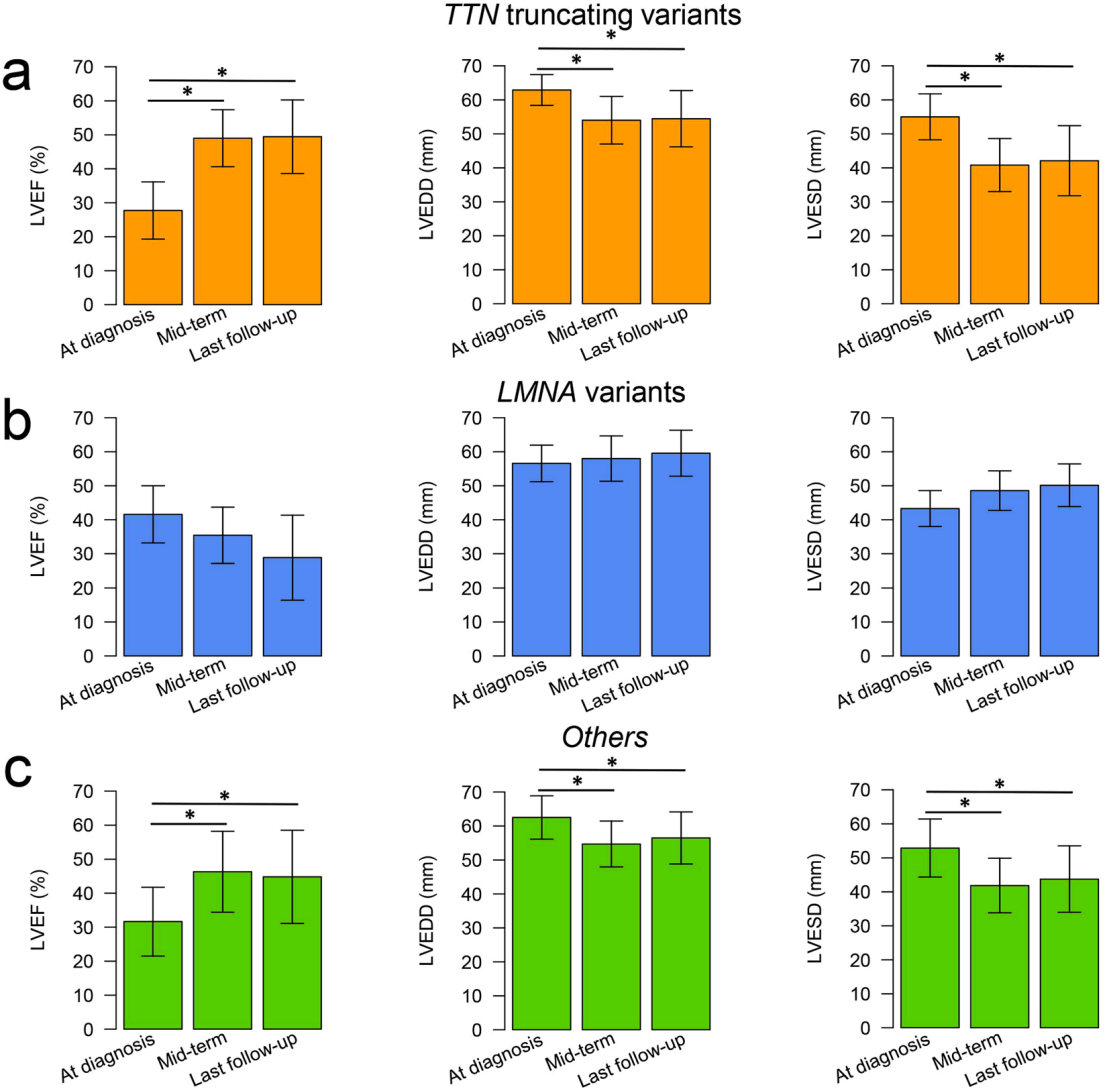
Changes in LVEF, LVEDD, and LVESD during follow-up in DCM patients harboring *TTN* truncating variants (n = 11), DCM patients harboring *LMNA* variants (n = 7), and other DCM patients (n = 27). The bar plot represents the changes in LVEF, LVEDD, and LVESD in patients with *TTN* truncating variants (**a**), patients with *LMNA* variants (**b**), and other patients **(c**) at diagnosis, mid-term (around 24 months), and last follow-up. Data are expressed as mean ± SD. *Represents *p* < 0.05 versus onset. DCM, dilated cardiomyopathy; LVEF, left ventricular ejection fraction; LVEDD, left ventricular end-diastolic diameter; LVESD, left ventricular end-systolic diameter.

LVRR was analyzed for 11 of 20 patients with *TTN* truncating variants, 7 of 13 patients with *LMNA* variants, and 27 of 87 other DCM patients. Most patients received standard medical therapy and there were no differences in treatment and dosage of beta-blockers among the three groups (Supplementary Table 8). LVRR was found in 9 patients (81.8%) with *TTN* truncating variants, in none of the patients with *LMNA* variants, and in 11 patients (40.7%) of other DCM patients (Table 2). In the *TTN* truncation group, LV ejection fraction (LVEF) was generally improved after initiation of therapy and was well maintained for a long period (Fig. 5a). In contrast to the *TTN* truncation group, LVEF in the *LMNA* group was not improved in most patients (Fig. 5b). In the other DCM patient group, the rate of LVRR was low compared with that in the *TTN* truncation group, but overall LVEF improved during follow-up (Fig. 5c). We also evaluated baseline characteristics and clinical information during follow-up of patients enrolled into LVRR analysis (Supplementary Table 9). As expected, the patients with LVRR had better prognosis, whereas 6 patients without LVRR reached the primary endpoint (heart transplantation and death) and they all had *LMNA* variants. Multivariate logistic regression analysis demonstrated that the *TTN* truncating variants and LVEF were independent factors for LVRR (Supplementary Table 10).

### Genotype–phenotype associations in HCM patients

Based on the results of the genetic analysis, HCM patients were divided into three groups consisting of those with *MYH7* variant, those with *MYBPC3* variant, and others, to investigate genotype–phenotype associations. Most patients in the *MYH7* group were female. Although the differences did not reach statistical significance, more patients had atrial fibrillation in the *MYBPC3* group and patients in the *MYH7* and *MYBPC3* groups were at higher risk of ICD or CRT-D implantation and end-stage HCM than were other patients (Supplementary Table 11). We found a novel recurrent *MYH7* p.E504G VUS in 2 unrelated patients as described above. Both patients had life-threatening arrhythmia and were hospitalized for worsening heart failure during follow-up, suggesting that the variant might be associated with worse prognosis.

## Discussion

Through integration of targeted sequencing and genotype-phenotype correlation analysis, we unveiled the genetic basis of cardiomyopathy and the genotypes involved in LVRR. In our DCM cohort, *TTN* variants were the most frequent variants identified, with *LMNA* variants the second-most frequent. In half of the HCM patients, *MYBPC3* and *MYH7* variants were responsible for the pathogenesis of HCM, consistent with previous reports^13,14^. We also revealed that patients with *TTN* truncating variants had better prognosis and responses to optimal therapy. In contrast, patients with *LMNA* variants had worse outcomes and poor responses to therapy. Such genetic screening contributes to our understanding of the genetic basis of DCM and HCM, and the prediction of prognosis in DCM.

The genetic profile of DCM in our Japanese cohort was different from that in Caucasian patients. In Caucasians, *TTN* truncating variants are most commonly responsible for familial DCM as well as sporadic DCM (19–25% and 11–18%, respectively)^7,15,16^. In our study, *TTN* truncating variants were the major cause of sporadic DCM (21.4% of sporadic cases) but not of familial DCM whereas *LMNA* variants were the most frequent variants in familial DCM (24.0% of familial cases). *TTN* truncating variants were the second-most frequent variants but were observed in only 10.0% of familial DCM patients in our study. Furthermore, we found a recurrent *LMNA* E115M variant that was shared by the 3 unrelated familial DCM patients (Supplementary Table 4). This recurrent E115M variant is not present in any population database and could be the Japanese founder mutation associated with DCM. Meanwhile, although Akinrinade *et al*. reported the *DSP* truncating mutation as a Finnish founder mutation in DCM^7^, we found only one *DSP* splice site variant in a single patient. These findings suggest that there are ethnic differences in the genetic profile of DCM.

Multiple rare variants might be associated with early disease onset or severe cardiomyopathies within a pedigree^17,18^. In this study, we found that 1 DCM patient who harbored multiple PMs, who was diagnosed at 25 years of age, developed a life-threatening arrhythmia, and was considered for heart transplantation, suggesting that multiple PMs would contribute to the severe phenotype of DCM. Meanwhile, the other patients with multiple variants, 1 PM and VUSs, or only VUSs, had no differences in prognosis from patients with single variant (Supplementary Figs 2 and 3), suggesting that the effects of multiple VUSs in DCM are still unclear. To accurately assess the overall genetic burden for each patient, weighting the contribution of each variant to disease severity might be helpful. Among HCM variants, for example, *MYH7* p. R719W was previously reported to be associated with severe HCM^19^. We found this variant in 2 unrelated patients with end-stage HCM (Supplementary Table 4). Furthermore, in this study, we found a novel recurrent *LMNA* E115M VUS in 3 unrelated DCM patients with a family history of sudden cardiac death or heart transplantation and a novel recurrent *MYH7* p.E504G VUS in 2 unrelated HCM patients with life-threatening arrhythmia who were hospitalized for heart failure. Although further studies are needed, the novel recurrent variants in this study might be the potent variants associated with worse prognosis.

Importantly, patients with *TTN* truncating variants tend to show LVRR after appropriate medical treatment. In the present study, LVRR was found in 20 of 45 patients (44.4%) in total, which is similar to previous reports^11,12^, and LVRR was observed in most patients (81.8%) with *TTN* truncating variants. Although patients harboring *TTN* truncating variants were likely to have low baseline LVEF (Fig. 5), which is the characteristic feature associated with LVRR^20,21^, *TTN* truncating variants are independently associated with LVRR (Supplementary Table 10). Collectively, although most probands with *TTN* truncating variants might be diagnosed when cardiac performance was impaired, they showed a good response to treatment and exhibited LVRR, leading to the better prognosis. In contrast to patients with *TTN* truncating variants, patients harboring *LMNA* variants showed high baseline LVEF without LVRR (Fig. 5). Although *LMNA* variants were not independently associated with LVRR in this study probably due to sample size restriction or the presence of confounding factors, our finding that no patients with *LMNA* variants exhibited LVRR might reflect the natural history of cardiomyopathy with *LMNA* mutation^22^. Thus, patients harboring *TTN* truncating variants would benefit from a precise genetic diagnosis followed by the appropriate medical therapy, while patients harboring *LMNA* variants should be followed up carefully and be considered for heart transplantation early.

There are several limitations in our study. First, although the present study was a multicenter study, all institutions participating in this study were highly advanced centers, and the patients may have been subjected to a selection bias. Particularly, the prevalence of end-stage HCM is higher than previously reported and the present findings may not readily be applied to HCM in general. Second, we retrospectively analyzed genotype–phenotype associations. We could not evaluate the LVRR of all the DCM patients due to the inherent nature of retrospective studies based on the data in clinical practice. Prospective studies and a larger number of cohorts are needed to confirm the genotype–phenotype associations discussed here.

**Figure 6:**
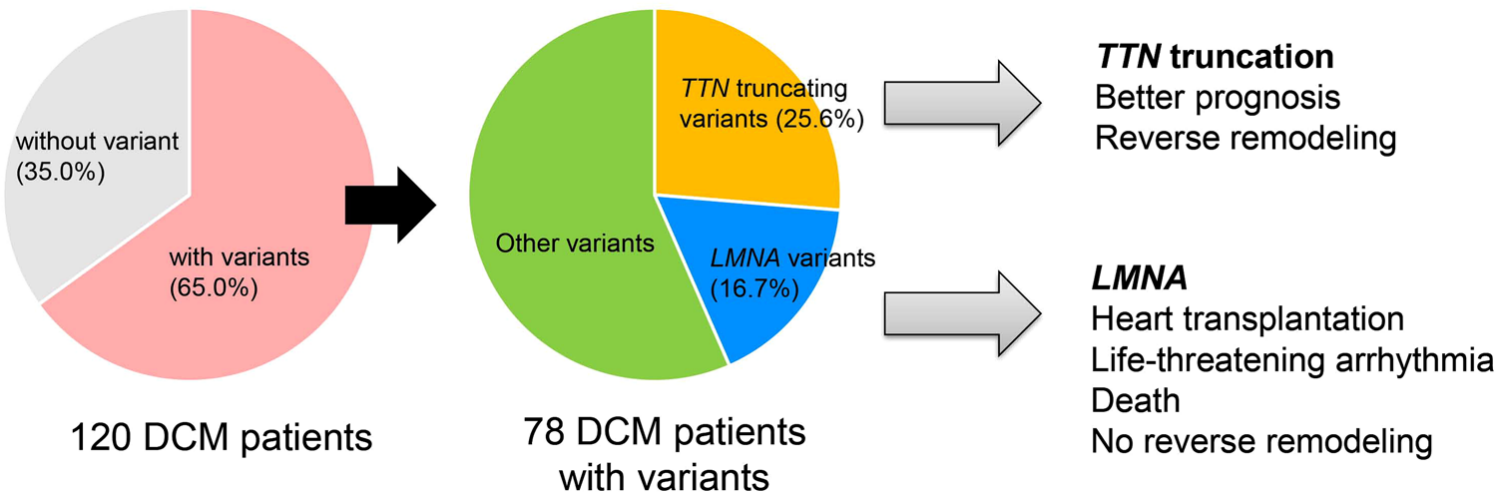
Genetic basis and genotypes involved in prognosis and left ventricular reverse remodeling of DCM patients. Genetic analysis was performed on 120 DCM patients. Among them, 78 (65.0%) patients had variants. *TTN* truncating variants were the most frequent. *TTN* truncating variants were associated with better prognosis and the presence of left ventricular reverse remodeling. *LMNA* variants were the second-most frequent. *LMNA* variants were associated with poor prognosis.

Our integrated analysis of target sequencing revealed the genetic basis and genotype-phenotype associations of cardiomyopathy in Japanese populations. DCM patients harboring *TTN* truncating variants likely exhibit LVRR and have better prognosis, whereas those with *LMNA* variants show poor response to medical therapy and are more likely to suffer from life-threatening arrhythmia and require heart transplantation (Fig. 6). These results suggest the potential application of this genetic information to the clinical setting.

## Materials and Methods

### Patients, cardiomyopathy definitions, and study design

This multicenter study was approved by the institutional review board of the participating institutions and conducted according to the guidelines of the Declaration of Helsinki. Informed consent was obtained from all patients (120 DCM patients and 52 HCM patients).

DCM and HCM were defined according to the commonly used WHO/International Society and Federation of Cardiology Task Force clinical criteria^23^. Briefly, DCM was defined as the presence of LV dilation (LV end-diastolic diameter [LVEDD] more than the average value of the healthy Japanese population corrected for age and sex^24^) and LV dysfunction (LVEF ≤50%) in the absence of abnormal loading conditions, such as hypertensive heart disease, primary valve disease, or significant coronary artery disease. HCM was defined as the presence of hypertrophy in any LV myocardial segment by two-dimensional echocardiography in the absence of dilated LV and another cardiac or systemic disease capable of producing the magnitude of ventricular hypertrophy^25,26^. End-stage HCM was defined as the presence of LVEF <50% during follow-up. Familial cardiomyopathy subjects were defined as patients with at least 1 additional affected family member with any cardiomyopathy or patients with family history of sudden cardiac death.

Clinical data were collected from physicians who were blinded to the genetic data. The primary endpoint was a composite outcome of death and heart transplantation and the secondary endpoint was life-threatening arrhythmia including sustained ventricular tachycardia, ventricular fibrillation, or cardiopulmonary resuscitation.

### Target enrichment, sequencing, and variant evaluations

Genomic DNA from all available individuals was extracted from whole blood samples by standard techniques. For this study, we designed a panel consisting of 19,636 amplicons for 95 genes (Supplementary Table 1) associated with DCM, HCM, and other inherited cardiovascular diseases using SureDesign for HaloPlex technology (Agilent Technologies Inc., Santa Clara, CA); 99.4% of the target regions were covered by the designed amplicons. Sequence library preparation for all subjects was performed according to the HaloPlex target enrichment system protocol for Illumina paired-end sequencing. Sequencing was performed on an Illumina HiSeq. 2500 instrument (Illumina Inc., San Diego, CA) in rapid run mode, producing 150-bp paired-end reads. FASTQ files were analyzed using SureCall, and all filtered reads were mapped to the human reference genome GRCh37/hg19 with BWA-MEM^27^. Initial detection of variants was carried out using SureCall, which comprises SAMtools^28^ and SNPPET (Agilent Technologies), with a minimum coverage of 20-fold. Then, we inspected the mapped reads and called variants on Integrative Genomics Viewer (IGV) to confirm the variants detected by the pipeline above, and excluded variants in introns and synonymous variants. We also excluded variants with an alternative allele frequency greater than 0.01% in any freely accessible population database in the ethnically matched 1000 Genomes database^29^, Exome Aggregation Consortium Browser^30^, Human Genetic Variation Database (HGVD, http://www.genome.med.kyoto-u.ac.jp/SnpDB), and ToMMo database^31^. All variants were predicted *in silico* using CADD scores^32^ and were excluded if CADD scores were less than 10. After variant filtering, variants were checked for known pathogenic relationships with cardiovascular diseases in the Human Genome Mutation Database (HGMD) Professional^33^. The variants were classified as PMs if they were in cardiomyopathy-related genes and previously reported as pathogenic in HGMD or predicted as truncating variants, including frameshift insertions or deletions, nonsense mutations, and splice site variants. Other variants were classified as VUSs (Fig. 1).

### Echocardiography and left ventricular reverse remodeling

Echocardiography results were confirmed by more than two cardiac echocardiography specialists. All procedures were performed according to the guidelines of the American Society of Echocardiography^34^. LVRR was defined as an absolute increase in LVEF of at least 10% with a final value of >35% or follow-up LVEF >50%, accompanied by a decrease in LVEDD of at least 10% or a final indexed LVEDD of <33 mm/m^2 ^^11,12^, as assessed by echocardiography at mid-term (around 24 months) after treatment. LVRR was also assessed in DCM patients who could be followed from diagnosis and who underwent echocardiography at mid-term after initiation of treatment for DCM. Patients who died, underwent heart transplantation or ventricular assist device implantation, or were followed up at another institution were excluded from the analysis of LVRR.

### Statistical analysis

Continuous and categorical data are expressed as the mean ± standard deviation (SD) or median (interquartile) and counts (percentages), respectively. Student’s *t*-test was used for continuous variables, and Fisher’s exact test was used for categorical variables. Survival curves were calculated by using the Kaplan–Meier method, and comparisons between curves were carried out by using log-rank tests. Step-wise multivariate logistic regression analysis was performed to identify the predictors of LVRR. Variables that differed significantly between DCM patients with and without LVRR in univariate analysis were entered into the multivariate analysis. Statistical analyses were performed using SAS software JMP version 11.0. Differences with a *p* value less than 0.05 were considered significant. For multiple comparisons across the three groups, differences with a Bonferroni-corrected *p* value less than 0.017 were considered significant.

### Data availability

All data generated or analyzed during this study are available from the corresponding authors upon reasonable request.

## Electronic supplementary material

Supplementary information
