# Supplementary material for: Genetic basis of cardiomyopathy and the genotypes involved in prognosis and left ventricular reverse remodeling
Source: Sci Rep. 2018 Jan 31;8:1998. doi: 10.1038/s41598-018-20114-9 (PMC5792481; doi:10.1038/s41598-018-20114-9)
Supplement: Supplementary file 1 — Supplementary information [file 41598_2018_20114_MOESM1_ESM.pdf]

Supplementary Materials

Genetic basis of cardiomyopathy and the genotypes involved in prognosis and left ventricular reverse remodeling

Takashige Tobita[1,2]*, Seitaro Nomura[3,4]*, Takanori Fujita[3], Hiroyuki Morita[4], Yoshihiro Asano[5], Kenji Onoue[6], Masamichi Ito[4], Yasushi Imai[7], Atsushi Suzuki[1], Toshiyuki Ko[4], Masahiro Satoh[8], Kanna Fujita[4], Atsuhiko T Naito[4], Yoshiyuki Furutani[9], Haruhiro Toko[4], Mutsuo Harada[4], Eisuke Amiya[4], Masaru Hatano[4], Eiki Takimoto[4], Tsuyoshi Shiga[1], Toshio Nakanishi[9], Yasushi Sakata[5], Minoru Ono[10], Yoshihiko Saito[6], Seiji Takashima[11], Nobuhisa Hagiwara[1], Hiroyuki Aburatani[3], and Issei Komuro[4]

[1]Department of Cardiology, Tokyo Women's Medical University

[2]Research Fellow of Japan Society for the Promotion of Science

[3]Genome Science Division, Research Center for Advanced Science and Technology, The University of Tokyo

[4]Department of Cardiovascular Medicine, Graduate School of Medicine, The University of Tokyo

[5]Department of Cardiovascular Medicine, Osaka University Graduate School of Medicine

[6]First Department of Internal Medicine, Nara Medical University

[7]Division of Cardiovascular Medicine, Jichi Medical University

[8]Department of Cardiovascular Medicine, Chiba University Graduate School of Medicine

[9]Department of Pediatric Cardiology, Tokyo Women's Medical University

[10]Department of Cardiovascular Surgery, Graduate School of Medicine, The University of Tokyo

[11]Department of Medical Biochemistry, Osaka University Graduate School of Medicine

* These authors contributed equally to this work.

Correspondence: Hiroyuki Aburatani and Issei Komuro

Addresses for correspondence:

Full name: Hiroyuki Aburatani

Postal address: 4-6-1 Komaba, Meguro-ku, Tokyo 153-8904, Japan

Telephone: +81-3-5452-5352 Fax number: +81-3-5452-5355

E-mail: haburata-tky@umin.ac.jp

Full name: Issei Komuro

Postal address: 7-3-1 Hongo, Bunkyo-ku, Tokyo 113-8655, Japan

Telephone: +81-3-5800-6526 Fax number: +81-3-3815-2087

E-mail: komuro-tky@umin.ac.jp

Supplementary Table 1. Genes analyzed in this study.

	Gene symbol			
**Cardiomyopathy reated genes**	*ABCC9*	*ACTC1*	*ACTN2*	*ANKRD1*
	BAG3	*CALR3*	*CAV3*	*CRYAB*
	CSRP3	*DES*	*DMD*	*DSC2*
	DSG2	*DSP*	*EMD*	*EYA4*
	FXN	*GLA*	*ILK*	*JPH2*
	JUP	*LAMP2*	*LDB3*	*LMNA*
	MYBPC3	*MYH6*	*MYH7*	*MYL2*
	MYL3	*MYOM1*	*MYOZ2*	*MYPN*
	NEBL	*NEXN*	*PDLIM3*	*PKP2*
	PLN	*PRKAG2*	*PTPN11*	*RAF1*
	RBM20	*RYR2*	*SCN5A*	*SGCD*
	TAZ	*TCAP*	*TGFB3*	*TMEM43*
	TMPO	*TNNC1*	*TNNI3*	*TNNT2*
	TPM1	*TTN*	*TTR*	*TXNRD2*
	VCL			
**Arrhythmia related genes**	*AKAP9*	*ANK2*	*CACNA1C*	*CACNB2*
	CASQ2	*GPD1L*	*KCNE1*	*KCNE2*
	KCNE3	*KCNH2*	*KCNJ2*	*KCNQ1*
	SCN1B	*SCN3B*	*SCN4B*	*SNTA1*
**Noonan syndrome related genes**	*BRAF*	*CBL*	*HRAS*	*KRAS*
	MAP2K1	*MAP2K2*	*NF1*	*NRAS*
	RIT1	*SHOC2*	*SOS1*	*SPREED1*
**Marfan syndrome related genes**	*ACTA2*	*CBS*	*COL3A1*	*FBN1*
	FBN2	*MYH11*	*SLC2A10*	*SMAD3*
	TGFBR1	*TGFBR2*		

Gene symbols analyzed in our study are shown.

Supplementary Table 2. List of genetic variants identified in DCM patients.

Gene	Chromosome	Genomic Location	Nucleotide Change	Amino Acid Change	RefSeq	Count	Variant type	HGMD	Variant class	CADD score
ACTN2	1	236902621	c.866G>A	p.R299H	NM_001103	1	missense	NA	VUS	34
AKAP9	7	91631254	c.2023A>G	p.S675G	NM_05751	1	missense	NA	VUS	22.9
ANK2	4	114264189	c.4139C>T	p.P1380L	NM_001148	1	missense	NA	VUS	34
BAG3	10	121429549	c.367C>T	p.R123*	NM_004281	1	nonsense	DM	PM	37
BAG3	10	121429668	c.486_487insCCAGCCT	p.P163fs	NM_004281	1	frameshift	NA	PM	33
CACNB2	10	18828645	c.1813C>T	p.R605C	NM_201590	1	missense	NA	VUS	31
CBS	21	44480624	c.1072G>T	p.V358L	NM_000071	1	missense	NA	VUS	14.83
DES	2	220283563	c.379C>A	p.127S	NM_001927	1	missense	NA	VUS	34
DES	2	220286086	c.1048C>T	p.R350W	NM_001927	2	missense	DM	PM	34
DES	2	220286282	c.1244G>A	p.R415Q	NM_001927	1	missense	NA	VUS	34
DMD	X	32305755	c.6181G>A	p.A2061T	NM_004006	1	missense	NA	VUS	12.8
DMD	X	32841504	c.265G>A	p.V89I	NM_004006	1	missense	NA	VUS	24.2
DSP	6	7562969	c.682A>G	p.I228V	NM_004415	1	missense	NA	VUS	23.1
DSP	6	7567585	c.1045-2A>G	p.A349_splice	NM_004415	1	splice site	NA	PM	25.1
DSP	6	7579748	c.3326_3328delAGA	p.1110del	NM_004415	1	inframe-del	NA	VUS	15.05
DSP	6	7582915	c.5420A>G	p.Q1807R	NM_004415	1	missense	NA	VUS	17.08
EMD	X	153607864	c.20T>C	p.L7P	NM_000117	1	missense	NA	VUS	22.8
FBN1	15	48755410	c.5093A>G	p.N1698S	NM_000138	1	missense	NA	VUS	14.18
FBN2	5	127648409	c.4796T>C	p.V1599A	NM_001999	1	missense	NA	VUS	25.6
FBN2	5	127671695	c.3709C>T	p.R1237C	NM_001999	1	missense	NA	VUS	26.6
FBN2	5	127681166	c.3100G>A	p.G1034S	NM_001999	1	missense	NA	VUS	34
JPH2	20	42815248	c.98A>C	p.K33T	NM_020433	1	missense	NA	VUS	26.2
JUP	17	39919472	c.1260C>G	p.C420W	NM_002230	1	missense	NA	VUS	24.4
KCNQ1	11	2797263	c.1664G>A	p.R555H	NM_000218	1	missense	DM	VUS	34
LMNA	1	156084954	c.242A>T	p.E81V	NM_170707	1	missense	NA	VUS	29.5
LMNA	1	156085053	c.343_344delGAinsAT	p.E115M	NM_170707	3	missense	NA	VUS	31
LMNA	1	156104248	c.568C>T	p.R190W	NM_170707	2	missense	DM	PM	35
LMNA	1	156104312	c.632A>G	p.Y211C	NM_170707	1	missense	NA	VUS	20.4
LMNA	1	156104629	c.673C>T	p.R225*	NM_170707	1	nonsense	DM	PM	38
LMNA	1	156104666	c.711_712delTG	p.F237fs	NM_170707	1	frameshift	NA	PM	35
LMNA	1	156105070	c.904_905delCT	p.L302fs	NM_170707	1	frameshift	NA	PM	34
LMNA	1	156105813	c.1058A>G	p.Q353R	NM_170707	1	missense	DM	PM	25.9
LMNA	1	156106119	c.1273_1274delGA	p.E425fs	NM_170707	1	frameshift	NA	PM	35
LMNA	1	156106166	c.1320delG	p.V440fs	NM_170707	1	frameshift	NA	PM	35
MAP2K2	19	4090636	c.1163G>A	p.R388Q	NM_030662	1	missense	NA	VUS	23.3
MYBPC3	11	47362581	c.1900G>T	p.V634F	NM_000256	1	missense	NA	VUS	33
MYH11	16	15812297	c.5172-2A>G	p.R1724_splice	NM_002474	1	splice site	NA	VUS	23.2
MYH11	16	15835450	c.2729G>A	p.R910Q	NM_002474	1	missense	NA	VUS	34
MYH6	14	23856862	c.4526A>T	p.E1509V	NM_002471	1	missense	NA	VUS	31
MYOM1	18	3100422	c.3578C>A	p.T1193K	NM_003803	1	missense	NA	VUS	32
MYOM1	18	3215053	c.169G>A	p.E57K	NM_003803	1	missense	NA	VUS	21.4

MYOZ2	4	120079222	c.292G>A	p.E98K	NM_016599	1	missense	NA	VUS	20.5
MYPN	10	69918324	c.1399G>A	p.E467K	NM_032578	1	missense	NA	VUS	20.8
NEBL	10	21104612	c.2183C>A	p.T728N	NM_006393	1	missense	NA	VUS	19.23
NEXN	1	78401616	c.1360A>G	p.K454E	NM_144573	1	missense	NA	VUS	21.8
NF1	17	29676156	c.7145C>A	p.P2382H	NM_000267	1	missense	NA	VUS	32
NF1	17	29701168	c.8452G>A	p.V2818M	NM_000267	1	missense	DM?	VUS	25.6
PKP2	12	33030874	c.940G>A	p.G314R	NM_004572	1	missense	NA	VUS	23.8
RBM20	10	112559581	c.1705G>A	p.A569T	NM_001134363	1	missense	NA	VUS	28.6
RBM20	10	112572055	c.1900C>T	p.R634W	NM_001134363	2	missense	DM	PM	25.4
RBM20	10	112572062	c.1907G>A	p.R636H	NM_001134363	1	missense	DM	PM	28.3
RBM20	10	112590905	c.3538C>A	p.H1180N	NM_001134363	1	missense	NA	VUS	29.4
RYR2	1	237798272	c.6672C>T	p.R2258C	NM_001035	1	missense	NA	VUS	35
RYR2	1	237875094	c.10280A>G	p.N3427S	NM_001035	1	missense	NA	VUS	24.2
RYR2	1	237949300	c.13292A>C	p.E4431A	NM_001035	1	missense	NA	VUS	11.72
RYR2	1	237961422	c.14042A>G	p.D4681G	NM_001035	1	missense	NA	VUS	25.8
SCN5A	3	38627529	c.2440C>T	p.R814W	NM_198056	1	missense	DM	PM	34
SCN5A	3	38651326	c.833A>G	p.H278R	NM_198056	1	missense	NA	VUS	23.4
SCN5A	3	38662334	c.611C>A	p.A204E	NM_198056	1	missense	NA	VUS	33
SMAD3	15	67457668	c.478A>G	p.I160V	NM_005902	1	missense	NA	VUS	16.34
SOS1	2	39213197	c.3770C>T	p.T1257I	NM_005633	1	missense	NA	VUS	14.78
TNNC1	3	52485853	c.224A>G	p.D75G	NM_003280	1	missense	NA	VUS	25
TNNT2	1	201332507	c.487G>A	p.E163K	NM_001001430	1	missense	DM	PM	34
TNNT2	1	201333481	c.404C>T	p.A135V	NM_001001430	1	missense	NA	VUS	23.8
TTN	2	179397217	c.104125C>T	p.R34709C	NM_001267550	1	missense	NA	VUS	24.6
TTN	2	179397979	c.103363C>T	p.R34455C	NM_001267550	1	missense	NA	VUS	24.0
TTN	2	179398606	c.102736C>T	p.R34246C	NM_001267550	1	missense	NA	VUS	23.2
TTN	2	179398978	c.102364G>T	p.G34122*	NM_001267550	1	nonsense	NA	PM	73
TTN	2	179398981	c.102361A>G	p.K34121E	NM_001267550	1	missense	NA	VUS	16.35
TTN	2	179399281	c.102061C>G	p.Q34021E	NM_001267550	1	missense	NA	VUS	13.42
TTN	2	179399484	c.101858C>G	p.S33953C	NM_001267550	1	missense	NA	VUS	16.29
TTN	2	179404141	c.98651C>G	p.S32884C	NM_001267550	1	missense	NA	VUS	19.33
TTN	2	179404142	c.98650_98651insT	p.S32884fs	NM_001267550	1	frameshift	NA	PM	63
TTN	2	179404516	c.98275delG	p.E32759fs	NM_001267550	1	frameshift	NA	PM	63
TTN	2	179406264	c.97540G>C	p.D32514H	NM_001267550	1	missense	NA	VUS	23.4
TTN	2	179408359	c.96341T>C	p.V32114A	NM_001267550	1	missense	NA	VUS	20.6
TTN	2	179408584	c.96287C>G	p.A32096G	NM_001267550	1	missense	NA	VUS	21.1
TTN	2	179408605	c.96266_95267insA	p.N32089fs	NM_001267550	1	frameshift	NA	PM	63
TTN	2	179412812	c.93541G>A	p.E31181K	NM_001267550	1	missense	NA	VUS	23.1
TTN	2	179419300	c.88774A>G	p.I29592V	NM_001267550	1	missense	NA	VUS	15.69
TTN	2	179424065	c.86775_86793delTGGTGTTGGTATACCAGCT	p.F28925fs	NM_001267550	1	frameshift	NA	PM	62
TTN	2	179424113	c.86742_86745delCTAT	p.}I28914fs	NM_001267550	1	frameshift	NA	PM	62
TTN	2	179424732	c.86113_86126delAATGCTCCCTGTCT	p.L28705fs	NM_001267550	1	frameshift	NA	PM	63
TTN	2	179426204	c.84655C>T	p.L28219F	NM_001267550	1	missense	NA	VUS	17.84
TTN	2	179431246	c.79613C>T	p.T26538I	NM_001267550	1	missense	NA	VUS	19.29
TTN	2	179432118	c.78741C>A	p.F26247L	NM_001267550	1	missense	NA	VUS	17.22

TTN	2	179435968	c.74891C>T	p.P24964L	NM_001267550	1	missense	NA	VUS	19.56
TTN	2	179437461	c.73397delA	p.H24466fs	NM_001267550	1	frameshift	NA	PM	61
TTN	2	179438625	c.72233delT	p.I24078fs	NM_001267550	1	frameshift	NA	PM	61
TTN	2	179439674	c.71184_71185insG	p.P23729fs	NM_001267550	1	frameshift	NA	PM	61
TTN	2	179440628	c.70231G>A	p.G23411S	NM_001267550	1	missense	NA	VUS	22.6
TTN	2	179446774	c.66322A>T	p.K22108*	NM_001267550	1	nonsense	NA	PM	63
TTN	2	179449396	c.64972G>C	p.G21658R	NM_001267550	1	missense	NA	VUS	18.69
TTN	2	179453500	c.62952G>A	p.W20984*	NM_001267550	1	nonsense	NA	PM	63
TTN	2	179453592	c.62860C>A	p.P20954T	NM_001267550	1	missense	NA	VUS	17.12
TTN	2	179455175	c.61276delC	p.L20426fs	NM_001267550	1	frameshift	NA	PM	56
TTN	2	179456409	c.60137A>G	p.Y20046C	NM_001267550	1	missense	NA	VUS	21.1
TTN	2	179458541	c.58471_58485delGAGGTGACCAAAGAT	p.19491_19495del	NM_001267550	1	inframe-del	NA	VUS	23
TTN	2	179460434	c.57646delA	p.I19246fs	NM_001267550	1	frameshift	NA	PM	55
TTN	2	179477147	c.50104_50105insG	p.D16702fs	NM_001267550	1	frameshift	NA	PM	53
TTN	2	179481515	c.48099_48100delTA	p.Y16033fs	NM_001267550	1	frameshift	NA	PM	48
TTN	2	179482614	c.47467G>T	p.D15823Y	NM_001267550	1	missense	NA	VUS	21.1
TTN	2	179484532	c.46507_46508delAA	p.15503fs	NM_001267550	1	frameshift	NA	PM	48
TTN	2	179500302	c.41748delA	p.E13916fs	NM_001267550	1	frameshift	NA	PM	38
TTN	2	179509319	c.40433A>G	p.E13478G	NM_001267550	1	missense	NA	VUS	22.2
TTN	2	179517966	c.38790A>T	p.K12930N	NM_001267550	1	missense	NA	VUS	22.2
TTN	2	179545860	c.33286C>T	p.R11096C	NM_001267550	1	missense	NA	VUS	23.1
TTN	2	179567186	c.30428A>C	p.D10143A	NM_001267550	1	missense	NA	VUS	23
TTN	2	179567366	c.30248G>A	p.R10083H	NM_001267550	1	missense	NA	VUS	23.8
TTN	2	179588603	c.21383G>A	p.R7128H	NM_001267550	1	missense	NA	VUS	22.5
TTN	2	179605842	c.12117delC	p.P4039fs	NM_001267550	1	frameshift	NA	PM	21.3
TTN	2	179606414	c.11546C>G	p.P3849R	NM_001267550	1	missense	NA	VUS	17.65
TTN	2	179621086	c.11117T>C	p.L3706P	NM_001267550	1	missense	NA	VUS	13.56
TTN	2	179621336	c.10867A>C	p.T3623P	NM_001267550	1	missense	NA	VUS	13.99
TTN	2	179634405	c.15103+1G>A	p.P2968_splice	NM_001267550	1	splice site	NA	PM	26.5
TTN	2	179642221	c.4571A>G	p.D1524G	NM_001267550	1	missense	NA	VUS	22.5
TTN	2	179650691	c.2254C>T	p.R752C	NM_001267550	1	missense	NA	VUS	23.3
TTN	2	179659218	c.1306G>A	p.A436T	NM_001267550	1	missense	NA	VUS	23.3

The gene symbol, chromosome, position, nucleotide change, amino acid change, RefSeq number of each variant, count, variant type, evaluation of HGMD, variant class, and CADD score are shown. DCM, dilated cardiomyopathy; DM, disease-causing mutation; HGMD, Human Genome Mutation Database; NA, not applicable; PM, pathogenic mutation; VUS, variant of uncertain significance.

Supplementary Table 3. List of genetic variants identified in HCM patients.

Gene	Chromosome	Genomic Location	Nucleotide Change	Amino Acid Change	RefSeq	Count	Variant type	HGMD	Variant class	CADD score
ACTN2	1	236917368	c.1961A>C	p.Q654P	NM_001103	1	missense	NA	VUS	28.1
CACNA1C	12	2705119	c.2743A>G	p.I915V	NM_000719	1	missense	NA	VUS	13.49
DMD	X	32404439	c.4662G>T	p.E1554D	NM_004006	1	missense	NA	VUS	22.9
DSP	6	7575601	c.2510A>C	p.Q837P	NM_004415	1	missense	NA	VUS	27.3
DSP	6	7578697	c.2986G>C	p.A996P	NM_004415	1	missense	NA	VUS	25.4
FBN2	5	127614470	c.7202G>A	p.S2401N	NM_001999	2	missense	NA	VUS	15.56
KCNQ1	11	2594110	c.544G>T	p.G272V	NM_000218	1	missense	DM	VUS	24.2
LMNA	1	156104248	c.568C>T	p.R190W	NM_170707	1	missense	DM	PM	35
LMNA	1	156107526	c.1690C>T	p.H564Y	NM_170707	1	missense	NA	VUS	22.5
MYBPC3	11	47355283	c.2996_3013delGCAAGCCCCGGCCTCAGG	p.999_1004del	NM_000256	1	inframe-del	NA	VUS	36
MYBPC3	11	47356592	c.2905+1G>A	p.Q969_splice	NM_000256	1	splice site	DM	PM	27.6
MYBPC3	11	47356663	c.2833_2834delCG	p.R945fs	NM_000256	1	frameshift	DM	PM	35
MYBPC3	11	47359276	c.2377_2378insG	p.E793fs	NM_000256	1	frameshift	NA	PM	35
MYBPC3	11	47361201	c.2067+1G>A	p.Q689_splice	NM_000256	2	splice site	DM	PM	25.2
MYBPC3	11	47363554	c.1780delT	p.S593fs	NM_000256	3	frameshift	DM	PM	35
MYBPC3	11	47364155	c.1597delC	p.Q533fs	NM_000256	1	frameshift	NA	PM	33
MYBPC3	11	47372845	c.237C>G	p.Y79*	NM_000256	1	nonsense	DM	PM	24.5
MYH11	16	15931951	c.159G>C	p.E53D	NM_002474	1	missense	NA	VUS	24.9
MYH7	14	23893235	c.2803G>A	p.E935K	NM_000257	1	missense	DM	PM	33
MYH7	14	23894111	c.2546T>C	p.M849T	NM_000257	1	missense	DM	PM	22.9
MYH7	14	23894153	c.2504A>C	p.K835T	NM_000257	1	missense	DM	PM	28
MYH7	14	23894969	c.2221G>C	p.G741R	NM_000257	1	missense	DM	PM	33
MYH7	14	23895180	c.2155C>T	p.R719W	NM_000257	2	missense	DM	PM	34
MYH7	14	23897776	c.1511A>G	p.E504G	NM_000257	2	missense	NA	VUS	27.4
MYH7	14	23898213	c.1358G>A	p.R453H	NM_000257	1	missense	DM	PM	35
MYH7	14	23898214	c.1357C>T	p.R453C	NM_000257	1	missense	DM	PM	33
MYH7	14	23899110	c.1012G>A	p.V338M	NM_000257	1	missense	DM ?	VUS	25
MYH7	14	23900677	c.746G>A	p.R249Q	NM_000257	1	missense	DM	PM	28.6
MYL2	12	111352091	c.173G>A	p.R58Q	NM_000432	1	missense	DM	PM	29.2
MYL3	3	46900970	c.476C>T	p.T159M	NM_000258	1	missense	NA	VUS	28.9
MYL3	3	46902192	c.281G>A	p.R94H	NM_000258	1	missense	DM	PM	25.5
PRKAG2	7	151573662	c.44C>T	p.S15F	NM_016203	1	missense	NA	VUS	23.3
RYR2	1	237862274	c.9077C>T	p.A3026V	NM_001035	1	missense	NA	VUS	23.3
RYR2	1	237944873	c.11889T>G	p.S3963R	NM_001035	1	missense	NA	VUS	24.2
SGCD	5	156186244	c.716C>T	p.A239V	NM_000337	1	missense	NA	VUS	24
SMAD3	15	67457668	c.478A>G	p.I160V	NM_005902	1	missense	NA	VUS	16.34
TGFBR2	3	30686329	c.185A>T	p.D62V	NM_003242	1	missense	NA	VUS	21.1
TNNI3	19	55665397	c.544_546delAAG	p.182del	NM_000363	2	inframe-del	DM	PM	20.1
TPM1	15	63336295	c.184G>A	p.E62K	NM_001018005	1	missense	NA	VUS	29.6
TTN	2	179425685	c.85174G>A	p.A28392T	NM_001267550	1	missense	NA	VUS	19.75

TTN	2	179431387	c.79472C>G	p.P26491R	NM_001267550	1	missense	NA	VUS	16.07
TTN	2	179435327	c.75532G>A	p.D25178N	NM_001267550	1	missense	NA	VUS	23.2
TTN	2	179482614	c.47467G>T	p.D15823Y	NM_001267550	1	missense	NA	VUS	21.1
TTN	2	179571316	c.29285C>T	p.T9762I	NM_001267550	1	missense	NA	VUS	18.71

The gene symbol, chromosome, position, nucleotide change, amino acid change, RefSeq number of each variant, count, variant type, evaluation of HGMD, variant class, and CADD score are shown.
DM, disease-causing mutation; HCM, hypertrophic cardiomyopathy; HGMD, Human Genome Mutation Database; NA, not applicable; PM, pathogenic mutation; VUS, variant of uncertain significance.

Supplementary Table 4. List of recurrent variants in DCM and HCM patients.

	Gene	Chromosome	Genomic Location	Nucleotide Change	Amino Acid Change	RefSeq	HGMD	Count
**DCM**	*DES*	2	220286086	c.1048C>T	p.R350W	NM_001927	Known	2
	LMNA	1	156104248	c.568C>T	p.R190W	NM_170707	Known	2
	RBM20	10	112572055	c.1900C>T	p.R634W	NM_001134363	Known	2
	LMNA	1	156085053	c.343_344delGAinsAT	p.E115M	NM_170707	Novel	3

	Gene	Chromosome	Genomic Location	Nucleotide Change	Amino Acid Change	RefSeq	HGMD	Count
**HCM**	*FBN2*	5	127614470	c.7202G>A	p.S2401N	NM_001999	Novel	2
	MYBPC3	11	47361201	c.2067+1G>A	p.Q689_splice	NM_000256	Known	2
	MYH7	14	23895180	c.2155C>T	p.R719W	NM_000257	Known	2
	MYH7	14	23897776	c.1511A>G	p.E504G	NM_000257	Novel	2
	TNNI3	19	55665397	c.544_546delAAG	p.182del	NM_000363	Known	2
	MYBPC3	11	47363554	c.1780delT	p.S593fs	NM_000256	Known	3

The gene symbols, chromosomes, positions, nucleotide changes, amino acid changes, RefSeq numbers of recurrent variants, known or novel variant in HGMD, and counts are shown.
DCM, dilated cardiomyopathy; HCM, hypertrophic cardiomyopathy; HGMD, Human Genome Mutation Database.

Supplementary Table 5. Total number of rare variants in each patient with DCM or HCM.

		DCM		HCM	
		PM positive patients (%)	PM+VUS positive patients (%)	PM positive patients (%)	PM+VUS positive patients (%)
Number of variants	n = 0	83 (69.2)	42 (35.0)	28 (53.8)	17 (32.7)
	n ≥ 1	37 (30.8)	78 (65.0)	24 (46.2)	35 (67.3)
	n ≥ 2	1 (0.8)	33 (27.5)	0	14 (26.9)
	n ≥ 3	0	8 (6.7)	0	2 (3.8)
	n ≥ 4	0	2 (1.7)	0	0

The total numbers of PMs or PMs and VUSs in each patient with DCM or HCM are shown.
DCM, dilated cardiomyopathy; HCM, hypertrophic cardiomyopathy;
PM, pathogenic mutation; VUS, variant of uncertain significance.

Supplementary Table 6. Variant class and phenotype associations in DCM patients.

	with PM (n = 37)	with VUS only (n = 41)	without variant (n = 42)	*p* value, PM vs VUS	*p* value, PM vs without variant	*p* value, VUS vs without variant
Age at diagnosis (years)	41.2 ± 11.7	37.0 ± 14.5	39.3 ± 15.2	0.207	0.599	0.557
Male *	35 (94.6%)	29 (70.7%)	35 (83.3%)	0.007	0.162	0.200
Familial	20 (54.1%)	19 (46.3%)	11 (26.2%)	0.651	0.020	0.070
Familial history of sudden death	9 (24.3%)	11 (26.8%)	3 (7.1%)	1.000	0.057	0.020
NYHA functional class ≥ 3 *	11/35 (31.4%)	29/39 (74.4%)	25/42 (59.5%)	<0.001	0.021	0.238
B-type natriuretic peptide (pg/ml)	235 (62-600)	548 (164-1473)	335 (104-1012)	0.029	0.128	0.485
Cardiac catheterization	36 (97.3%)	40 (97.6%)	41 (97.6%)	1.000	0.474	1.000
Endomyocardial biopsy (n = 96)						
Inflammation	3/29 (10.3%)	8/33 (24.2%)	6/34 (17.6%)	0.194	0.488	0.560
Fibrosis	26/29 (89.7%)	29/33 (87.9%)	31/34 (91.2%)	1.000	1.000	0.709
Echocardiography (n = 113)						
LVEF (%)	28.9 ± 10.5	29.0 ± 12.4	31.6 ± 14.1	0.987	0.695	0.554
LVEDD (mm)	63.9 ± 8.3	68.1 ± 12.5	66.3 ± 12.5	0.123	0.324	0.506
LVESD (mm)	55.3 ± 10.6	60.3 ± 15.0	57.7 ± 15.1	0.134	0.423	0.472
IVST (mm)	7.7 ± 2.1	7.3 ± 2.2	8.4 ± 2.1	0.406	0.130	0.026
PWT (mm)	8.0 ± 2.1	7.4 ± 2.7	8.2 ± 2.4	0.233	0.681	0.132
LV mass (g)	211.6 ± 68.5	216.1 ± 102.0	240.5 ± 90.7	0.710	0.245	0.172
LAD (mm)	44.1 ± 8.5	45.3 ± 10.5	43.9 ± 9.8	0.508	0.773	0.387
Restrictive mitral pattern (%)	6/22 (27.3%)	14/29 (48.3%)	10/23 (43.4%)	0.157	0.353	0.785
E/e'	13.2 ± 8.5	15.8 ± 9.0	14.0 ± 12.2	0.352	0.819	0.184
Mitral regurgitation ≥ moderate	10/31 (32.3%)	18/39 (46.2%)	9/38 (23.7%)	0.327	0.589	0.056
Left ventricular reverse remodeling	11/21 (52.4%)	4/12 (33.3%)	5/12 (41.7%)	0.469	0.721	1.000
Cardiopulmonary exercise testing (n = 42)						
Rest exercise heart rate (beats/min)	76 ± 13	78 ± 14	85 ± 18	0.586	0.057	0.211
Peak exercise heart rate (beats/min)	130 ± 27	124 ± 33	121 ± 28	0.738	0.492	0.892
Rest exercise systolic blood pressure (mmHg)	98 ± 15	91 ± 20	94 ± 19	0.089	0.289	0.663
Peak exercise systolic blood pressure (mmHg) *	144 ± 30	115 ± 26	125 ± 31	0.011	0.151	0.562
Peak VO2 (mL/kg/min)	18.0 ± 6.9	13.2 ± 6.0	13.9 ± 5.9	0.017	0.069	0.935
Follow-up data						
Amiodarone	23 (62.2%)	16 (39.0%)	18 (42.9%)	0.069	0.115	0.824
Pacemaker implantation	1 (2.7%)	0	1 (2.4%)	0.474	1.000	1.000

ICD implantation	4 (10.8%)	3 (7.3%)	7 (16.7%)	0.702	0.528	0.313
CRT-D implantation †	17 (45.9%)	11 (26.8%)	8 (19.0%)	0.100	0.015	0.443
ICD or CRT-D implantation	21 (56.8%)	14 (34.1%)	15 (35.7%)	0.068	0.073	1.000
Any device	22 (59.5%)	14 (34.1%)	18 (42.9%)	0.040	0.178	0.501
AF	14 (37.8%)	11 (26.8%)	11 (26.2%)	0.338	0.335	1.000
Non-sustained VT	21 (56.8%)	21 (51.2%)	17 (40.5%)	0.656	0.179	0.382
Sustained VT	10 (27.0%)	10 (24.4%)	10 (23.8%)	0.802	0.799	1.000
VF, CPR	4 (10.8%)	7 (17.1%)	6 (14.3%)	0.524	0.743	0.771
Heart transplantation	4 (10.8%)	8 (19.5%)	10 (23.8%)	0.356	0.152	0.791
Mortality	4 (10.8%)	3 (7.3%)	4 (9.5%)	0.702	1.000	1.000
Heart transplantation or Mortality	8 (21.6%)	11 (26.8%)	14 (33.3%)	0.610	0.317	0.634
Mean follow-up duration (years)	8.5 ± 7.0	9.0 ± 9.0	8.6 ± 8.9	0.904	0.522	0.600

Values are n (%), the mean ± SD, or median (interquartile). Superscript letters represent significant differences compared with other groups (* = PM group versus VUS group; † = PM group versus without variant group).

DCM, dilated cardiomyopathy; NA, not applicable; NYHA, New York Heart Association;
LV, left ventricular; EF, ejection fraction; EDD, end-diastolic diameter;
ESD, end-systolic diameter; LAD, left atrial dimension;
ICD, implantable cardioverter defibrillator; VF, ventricular fibrillation;
CRTD, cardiac resynchronization therapy defibrillator; AF, atrial fibrillation;
VT, ventricular tachycardia; CPR, cardiopulmonary ; resuscitation; NA, not applicable.
PM, pathogenic mutation; VUS, variant of uncertain significance.

Supplementary Table 7. Variant class and phenotype associations in HCM patients.

	with PM (n = 24)	with VUS only (n = 11)	without variant (n = 17)	*p* value, PM vs VUS	*p* value, PM vs without variant	*p* value, VUS vs without variant
Age at diagnosis (years)	34.3 ± 17.4	24.1 ± 15.8	31.9 ± 17.1	0.126	0.662	0.239
Male	16 (66.7%)	6 (54.5%)	10 (58.8%)	0.708	0.745	1.000
Familial	16 (66.7%)	10 (90.9%)	11 (64.7%)	0.217	1.000	0.191
Familial history of sudden death	9 (37.5%)	5 (45.5%)	4 (23.5%)	0.721	0.499	0.409
NYHA functional class ≥ 3	10 (41.7%)	6 (54.5%)	4 (23.5%)	0.716	0.321	0.125
B-type natriuretic peptide (pg/ml)	339 (118-759)	658 (97-1230)	240 (91-903)	0.513	0.679	0.304
Cardiac catheterization	20 (83.3%)	9 (81.8%)	16 (94.1%)	1.000	0.382	0.543
Endomyocardial biopsy (n = 38)						
Inflammation	1/19 (5.3%)	0/7 (0%)	2/12 (16.7%)	1.000	0.544	0.509
Fibrosis	19/19 (100%)	7/7 (100%)	11/12 (91.7%)	NA	0.387	1.000
Echocardiography (n = 48)						
LVEF (%)	47.8 ± 15.9	45.5 ± 19.3	58.7 ± 25.5	0.655	0.056	0.196
LVEDD (mm)	53.6 ± 9.8	51.5 ± 13.7	50.8 ± 17.7	0.320	0.080	0.440
LVESD (mm)	41.0 ± 12.3	38.3 ± 18.8	36.6 ± 23.3	0.375	0.080	0.356
IVST (mm)	12.7 ± 4.3	12.1 ± 4.1	14 ± 4.8	0.734	0.318	0.302
PWT (mm)	9.7 ± 3.0	9.3 ± 3.1	9.1 ± 2.3	0.551	0.653	0.811
LV mass (g)	235.8 ± 75.9	207.9 ± 86.7	230.0 ± 142.8	0.245	0.209	0.712
LAD (mm)	46.0 ± 10.5	47.8 ± 10.5	39.4 ± 9.6	0.751	0.057	0.051
Restrictive mitral pattern (%)	7/19 (36.8%)	2/7 (28.6%)	3/15 (20.0%)	1.000	0.451	1.000
E/e'	14.3 ± 6.6	11.1 ± 5.3	14.4 ± 10.2	0.314	0.544	0.874
Mitral regurgitation ≥ moderate	3/21 (14.3%)	1/10 (10.0%)	3/16 (18.8%)	1.000	1.000	1.000
Maximum wall thickness	13.3 ± 4.2	13.0 ± 4.1	14.6 ± 5.3	0.825	0.322	0.315
Peak LVOT gradient ≥ 30mmHg	3/22 (13.6%)	0/10 (0%)	2/16 (12.5%)	0.534	1.000	0.508
Cardiopulmonary exercise testing (n = 18)						
Rest exercise heart rate (beats/min)	73 ± 10	69 ± 9	67.3 ± 6.4	0.477	0.550	1.000
Peak exercise heart rate (beats/min)	103 ± 30	104 ± 33	107 ± 32	1.000	1.000	1.000
Rest exercise systolic blood pressure (mmHg)	103 ± 18	82 ± 6	81 ± 11	0.040	0.076	1.000
Peak exercise systolic blood pressure (mmHg)	133 ± 33	114 ± 22	100 ± 21	0.472	0.073	0.596
Peak VO2 (mL/kg/min)	12.0 ± 2.9	10.6 ± 3.6	12.2 ± 2.6	0.267	1.000	0.596
Follow-up data						
Amiodarone	17 (70.8%)	5 (45.5%)	6 (35.3%)	0.258	0.031	0.701
Pacemaker implantation	0	0	0	NA	NA	NA
ICD implantation	3 (12.5%)	4 (36.4%)	3 (17.6%)	0.172	0.679	0.381

CRT-D implantation *	14 (58.3%)	2 (18.2%)	1 (5.9%)	0.035	<0.001	0.543
ICD or CRT-D implantation *	17 (70.8%)	6 (54.5%)	4 (23.5%)	0.451	0.004	0.125
Any device *	17 (70.8%)	6 (54.5%)	5 (29.4%)	0.451	0.012	0.248
AF *	15 (62.5%)	5 (45.5%)	2 (11.8%)	0.467	0.001	0.076
Non-sustained VT	11 (45.8%)	6 (54.5%)	3 (17.6%)	0.725	0.096	0.095
Sustained VT	5 (20.8%)	3 (27.3%)	1 (5.9%)	0.686	0.373	0.269
VF, CPR	3 (12.5%)	3 (27.3%)	3 (17.6%)	0.352	0.679	0.653
End-stage HCM (LVEF < 50%)	18 (75.0%)	7 (63.6%)	6 (35.3%)	0.689	0.023	0.246
Heart transplantation	2 (8.3%)	1 (9.1%)	3 (17.6%)	1.000	0.633	1.000
Mortality	1 (4.2%)	0	0	1.000	1.000	NA
Heart transplantation or Mortality	3 (12.5%)	1 (9.1%)	3 (17.6%)	1.000	0.679	1.000
Mean follow-up duration (years) †	18.0 ± 12.5	26.6 ± 10.2	9.7 ± 7.5	0.041	0.028	<0.001

Values are n (%), the mean ± SD, or median (interquartile). Superscript letters represent significant differences compared other groups
(* = PM group versus without variant group; † = VUS group versus without variant group.)

HCM, hypertrophic cardiomyopathy; NYHA, New York Heart Association;
LV, left ventricular; EF, ejection fraction; EDD, end-diastolic diameter;
ESD, end-systolic diameter; LAD, left atrial dimension;
LVOT, left ventricular outflow tract;
ICD, implantable cardioverter defibrillator; VF, ventricular fibrillation;
CRTD, cardiac resynchronization therapy defibrillator; AF, atrial fibrillation;
VT, ventricular tachycardia; CPR, cardiopulmonary ; resuscitation; NA, not applicable.
PM, pathogenic mutation; VUS, variant of uncertain significance.

Supplementary Table 8. Medication use in DCM patietns.

	TTN (n = 11)	*LMNA* (n = 7)	Others (n = 27)	*p* value, *TTN* vs *LMNA*	*p* value, *TTN* vs Others	*p* value, *LMNA* vs Others
Beta-blocker	11 (100%)	7 (100%)	26 (96.3%)	NA	1.000	1.000
Carvedilol equivalent dose (mg/day)	17.3 ± 10.9	12.5 ± 7.9	15.5 ± 11.0	0.459	0.559	0.763
ACEI/ ARB	11 (100%)	7 (100%)	26 (96.3%)	NA	1.000	1.000
Mineralocorticoid recepter antagonists	8 (72.7%)	5 (71.4%)	18 (66.7%)	1.000	1.000	1.000
Diuretics	9 (81.8%)	4 (57.1%)	17 (63.0%)	0.326	0.444	1.000

Values are n (%) or the mean ± SD. There were no significant differences compared with other groups.
DCM, dilated cardiomyopathy; ACEI, angiotensin converting enzyme inhibitors;
ARB, angiotensin receptor blockers; NA, not applicable.

Supplementary Table 9. Clinical features at baseline and clinical information during follow-up in DCM patients enrolled for LVRR analysis

	Total (n = 45)	LVRR present (n = 20)	LVRR absent (n = 25)	*p* value
Age at diagnosis (years)	41.6 ± 10.7	46.2 ± 10.3	38.0 ± 9.7	0.016
Male	37 (82.2%)	17 (85.0%)	20 (80.0%)	0.716
Familial	22 (48.9%)	8 (40.0%)	14 (56.0%)	0.373
Familial history of sudden death	10 (22.2%)	2 (10.0%)	8 (32.0%)	0.147
NYHA functional class ≥ 3	19 (42.2%)	10 (50.0%)	9 (36.0%)	0.379
B-type natriuretic peptide (pg/ml)	203 (44-770)	371 (132-978)	105 (20-499)	0.039
Cardiac catheterization	44 (97.8%)	20 (100%)	24 (96.0%)	1.000
TTN truncating variant	11 (24.4%)	9 (45.0%)	2 (8.0%)	0.006
LMNA variants	7 (15.6%)	0	7 (28.0%)	0.012
Endomyocardial biopsy (n = 36)				
Inflammation	4/36 (11.1%)	1/17 (5.9%)	3/19 (15.8%)	0.605
Fibrosis	31/36 (86.1%)	15/17 (88.2%)	16/19 (84.2%)	1.000
Echocardiography				
LVEF (%)	32.2 ± 10.3	25.6 ± 6.5	37.5 ± 9.7	<0.0001
LVEDD (mm)	61.7 ± 6.1	64.2 ± 6.0	59.7 ± 5.6	0.029
LVESD (mm)	51.9 ± 8.5	56.4 ± 6.9	48.3 ± 8.0	<0.001
IVST (mm)	8.6 ± 2.1	8.4 ± 1.9	8.8 ± 2.3	0.770
PWT (mm)	9.0 ± 1.9	9.2 ± 1.7	9.0 ± 2.1	0.623
LV mass (g)	228.1 ± 74.6	242.2 ± 78.7	215.9 ± 70.5	0.374
LAD (mm)	41.9 ± 7.2	42.9 ± 6.9	41.1 ± 7.5	0.295
Restrictive mitral pattern (%)	8/27 (29.6%)	6/13 (46.1%)	2/14 (14.3%)	0.103
E/e'	10.7 ± 6.1	12.4 ± 7.6	8.9 ± 3.2	0.166
Mitral regurgitation ≥ moderate	9/40 (22.5%)	5/19 (26.3%)	4/21 (19.0%)	0.712
Cardiopulmonary exercise testing (n = 13)				
Rest exercise heart rate (beats/min)	73 ± 11	77 ± 9	69 ± 11	0.197
Peak exercise heart rate (beats/min)	139 ± 20	142 ± 13	136 ± 28	1.000
Rest exercise systolic blood pressure (mmHg)	97 ± 17	97 ± 16	97 ± 20	0.829
Peak exercise systolic blood pressure (mmHg)	151 ± 29	155 ± 14	145 ± 41	0.317
Peak VO2 (mL/kg/min)	20.1 ±7.3	20.8 ± 3.0	19.4 ± 10.7	0.352
Follow-up data				
Amiodarone	25 (55.6%)	12 (60.0%)	13 (52.0%)	0.764
Pacemaker implantation	0	0	0	NA

ICD implantation	8 (17.8%)	5 (25.0%)	3 (12.0%)	0.435
CRT-D implantation	8 (17.8%)	2 (10.0%)	6 (24.0%)	0.269
ICD or CRT-D implantation	16 (35.6%)	7 (35.0%)	9 (36.0%)	1.000
Any device	16 (35.6%)	7 (35.0%)	9 (36.0%)	1.000
AF	14 (31.1%)	5 (25.0%)	9 (36.0%)	0.526
Non-sustained VT	25 (55.6%)	13 (65.0%)	12 (48.0%)	0.367
Sustained VT	11 (24.4%)	5 (25.0%)	6 (24.0%)	1.000
VF, CPR	4 (8.9%)	3 (15.0%)	1 (4.0%)	0.309
Heart transplantation	3 (6.7%)	0	3 (12.0%)	0.242
Mortality	3 (6.7%)	0	3 (12.0%)	0.242
Heart transplantation or Mortality	6 (13.3%)	0	6 (24.0%)	0.027
Mean duration to mid-term (years)	2.1 ± 0.6	1.9 ± 0.5	2.2 ± 0.6	0.043
Mean follow-up duration (years)	5.2 ± 3.6	4.3 ± 3.0	6.0 ± 3.9	0.167

Values are n (%), the mean ± SD, or median (interquartile).
DCM, dilated cardiomyopathy; NYHA, New York Heart Association;
LV, left ventricular; EF, ejection fraction; EDD, end-diastolic diameter;
ESD, end-systolic diameter; LAD, left atrial dimension;
ICD, implantable cardioverter defibrillator; VF, ventricular fibrillation;
CRTD, cardiac resynchronization therapy defibrillator; AF, atrial fibrillation;
VT, ventricular tachycardia; CPR, cardiopulmonary ; resuscitation; NA, not applicable.

Supplementary Table 10. Independent predictors of LVRR.

	OR	95% CI	*p* value
TTN truncating variants	10.69	1.66-109.3	0.011
LVEF	0.86	0.76-0.94	<0.001

OR estimation is referred to every unit increase or decrease for continuous variables.
CI, confidence interval; LVRR, left ventricular reverse remodeling; OR, odds raio.

Supplementary Table 11. Genotype–phenotype associations in HCM patients.

	MYH7 (n = 12)	*MYBPC3* (n = 11)	Others (n = 29)	*p* value, *MYH7* vs *MYBPC3*	*p* value, *MYH7* vs Others	*p* value, *MYBPC3* vs Others
Age at diagnosis (years)	25.4 ± 17.6	39.0 ± 12.6	30.9 ± 17.8	0.049	0.344	0.150
Male *	3 (25.0%)	10 (90.9%)	19 (65.5%)	0.003	0.037	0.233
Familial	9 (75.0%)	7 (63.6%)	21 (72.4%)	0.667	1.000	0.704
Familial history of sudden death	5 (41.7%)	2 (18.2%)	11 (37.9%)	0.371	1.000	0.286
NYHA functional class ≥ 3	7 (58.3%)	5 (45.5%)	8 (27.6%)	0.684	0.083	0.451
B-type natriuretic peptide (pg/ml)	419 (129-685)	362 (110-865)	282 (109-943)	0.820	0.723	0.942
Cardiac catheterization	11 (91.7%)	8 (72.7%)	26 (89.7%)	0.317	1.000	0.319
Endomyocardial biopsy (n = 38)						
Inflammation	1/10 (10.0%)	0/8 (0%)	2/20 (10.0%)	1.000	1.000	1.000
Fibrosis	10/10 (100%)	8/8 (100%)	19/20 (95.0%)	NA	1.000	1.000
Echocardiography (n = 48)						
LVEF (%)	48.8 ± 18.1	44.3 ± 16.0	54.1 ± 22.8	0.500	0.493	0.201
LVEDD (mm)	51.8 ± 11.9	52.7 ± 9.0	52.2 ± 15.6	0.879	0.711	0.380
LVESD (mm)	39.9 ± 15.6	40.0 ± 11.2	38.1 ± 20.6	1.000	0.479	0.257
IVST (mm)	11.1 ± 3.5	13.8 ± 4.6	13.5 ± 4.6	0.252	0.103	0.956
PWT (mm)	8.5 ± 2.2	10.1 ± 2.8	9.5 ± 3.0	0.249	0.464	0.462
LV mass (g)	186.6 ± 65.0	252.2 ± 74.1	236.6 ± 121.2	0.058	0.157	0.306
LAD (mm)	43.8 ± 10.5	47.6 ± 8.7	43.1 ± 11.3	0.322	0.809	0.148
Restrictive mitral pattern (%)	3/8 (37.5%)	2/7 (28.6%)	7/26 (26.9%)	1.000	0.666	1.000
E/e'	12.3 ± 5.9	15.0 ± 7.7	13.7 ± 8.7	0.609	0.638	0.561
Mitral regurgitation ≥ moderate	0/11 (0%)	2/9 (22.2%)	5/27 (18.5%)	0.190	0.295	1.000
Maximum wall thickness	11.6 ± 3.6	14.6 ± 4.0	14.3 ± 4.9	0.169	0.069	1.000
Peak LVOT gradient ≥ 30mmHg	2/12 (16.7%)	0/9 (0%)	3/27 (11.1%)	0.486	0.634	0.558
Cardiopulmonary exercise testing (n = 18)						
Rest exercise heart rate (beats/min)	75 ± 5	68 ± 8	71 ± 11	0.298	0.411	0.745
Peak exercise heart rate (beats/min)	112 ± 24	94 ± 31	110 ± 30	0.255	0.919	0.385
Rest exercise systolic blood pressure (mmHg)	105 ± 13	102 ± 23	84 ± 10	0.699	0.053	0.107
Peak exercise systolic blood pressure (mmHg)	110 ± 14	144 ± 37	110 ± 20	0.255	1.000	0.064
Peak VO2 (mL/kg/min)	11.0 ± 4.1	12.4 ± 2.7	11.5 ± 2.9	1.000	0.919	0.452
Follow-up data						
Amiodarone	6 (50.0%)	7 (63.6%)	15 (51.7%)	0.680	1.000	0.724
Pacemaker implantation	0	0	0	NA	NA	NA
ICD implantation	2 (16.7%)	2 (18.2%)	6 (20.7%)	1.000	1.000	1.000
CRT-D implantation	6 (50.0%)	6 (54.5%)	5 (17.2%)	1.000	0.052	0.042
ICD or CRT-D implantation	8 (66.7%)	8 (72.7%)	11 (37.9%)	1.000	0.168	0.078

Any device	8 (66.7%)	8 (72.7%)	12 (41.4%)	1.000	0.181	0.155
AF	5 (41.7%)	8 (72.7%)	9 (31.0%)	0.214	0.719	0.031
Non-sustained VT	3 (25.0%)	6 (54.5%)	11 (37.9%)	0.214	0.494	0.477
Sustained VT	2 (16.7%)	4 (36.4%)	3 (10.3%)	0.371	0.620	0.076
VF, CPR	3 (25.0%)	2 (18.2%)	4 (13.8%)	1.000	0.398	1.000
End-stage HCM (LVEF < 50%)	8 (66.7%)	8 (72.7%)	15 (51.7%)	1.000	0.497	0.297
Heart transplantation	1 (8.3%)	1 (9.1%)	4 (13.8%)	1.000	1.000	1.000
Mortality	1 (8.3%)	0	0	1.000	0.293	NA
Heart transplantation or Mortality	2 (16.7%)	1 (9.1%)	4 (13.8%)	1.000	1.000	1.000
Mean follow-up duration (years)	20.1 ± 11.3	15.8 ± 11.2	16.4 ± 12.9	0.355	0.315	0.952

Values are n (%), the mean ± SD, or median (interquartile). Superscript letters represent significant differences compared with other groups (* = *MYH7* group versus *MYBPC3* group).

HCM, hypertrophic cardiomyopathy; NYHA, New York Heart Association;
LV, left ventricular; EF, ejection fraction; EDD, end-diastolic diameter;
ESD, end-systolic diameter; LAD, left atrial dimension;
LVOT, left ventricular outflow tract;
ICD, implantable cardioverter defibrillator; VF, ventricular fibrillation;
CRTD, cardiac resynchronization therapy defibrillator; AF, atrial fibrillation;
VT, ventricular tachycardia; CPR, cardiopulmonary ; resuscitation; NA, not applicable.

Supplementary Figure 1.

a

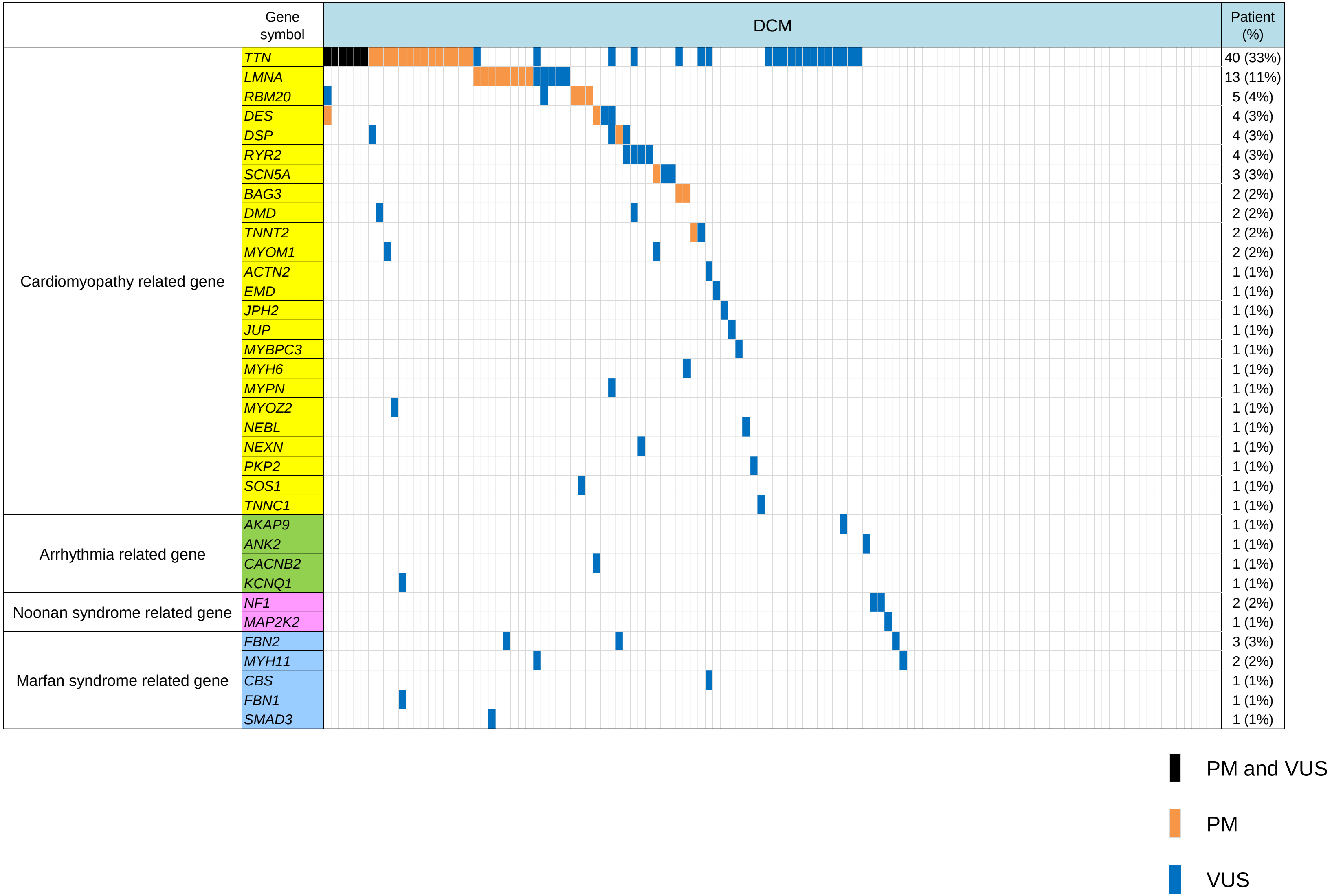

Supplementary Figure 1.

b

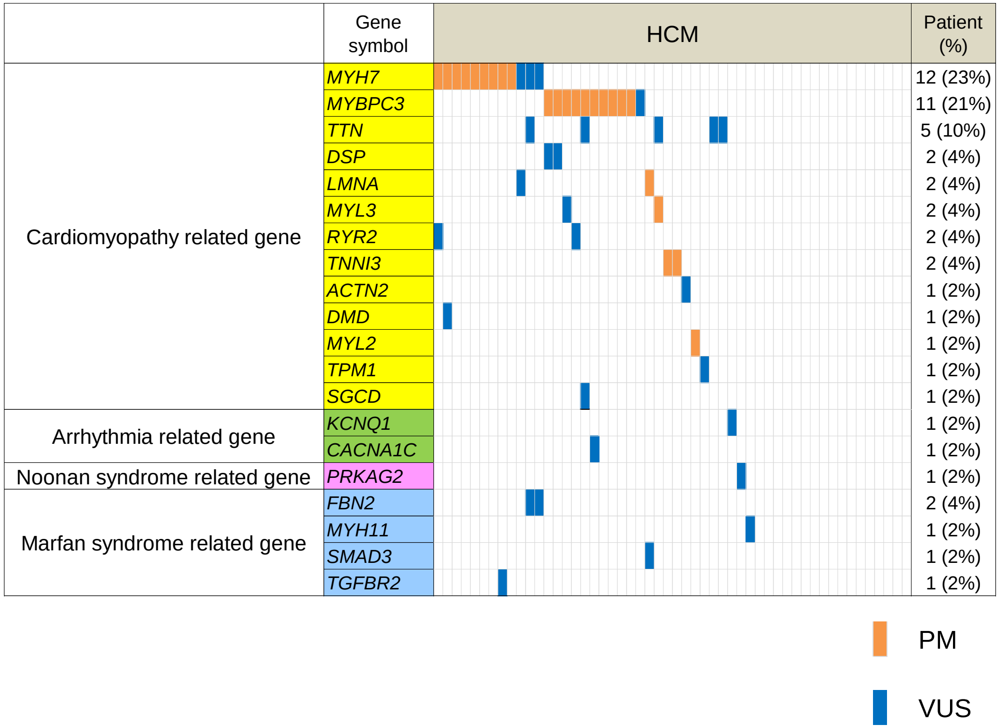

Supplementary Figure 2.

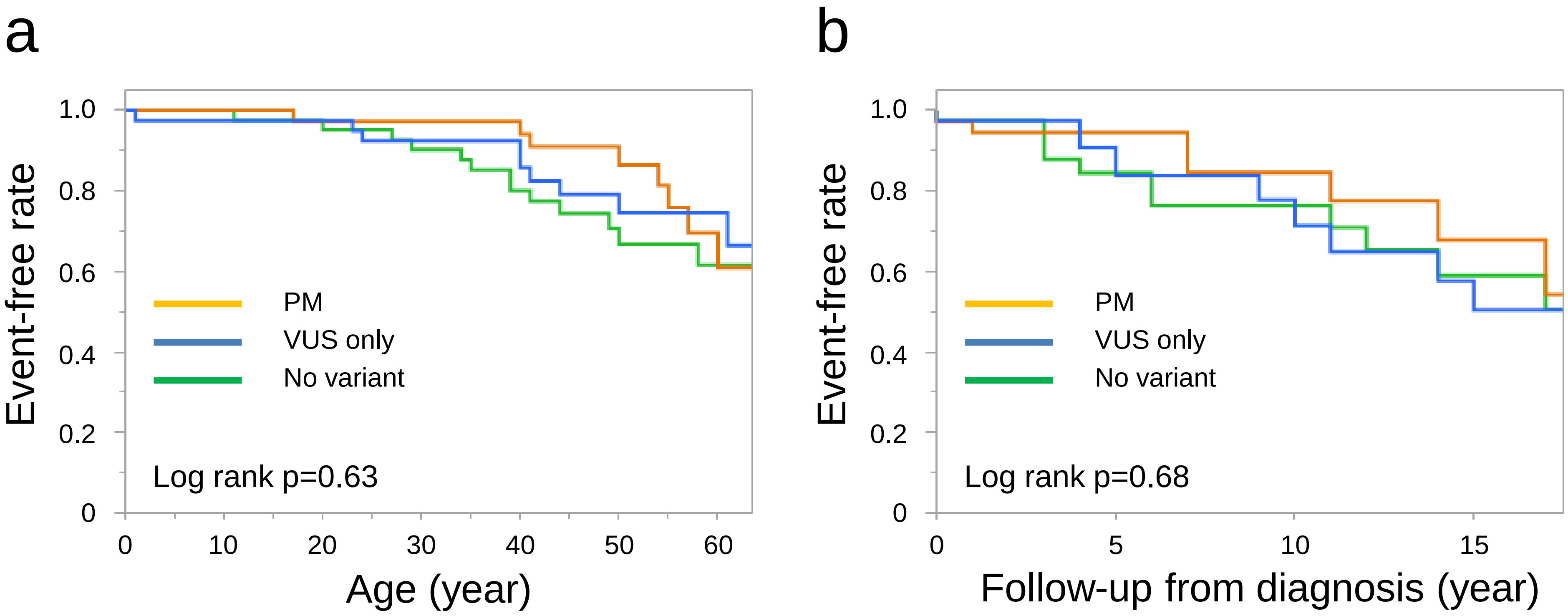

Supplementary Figure 3.

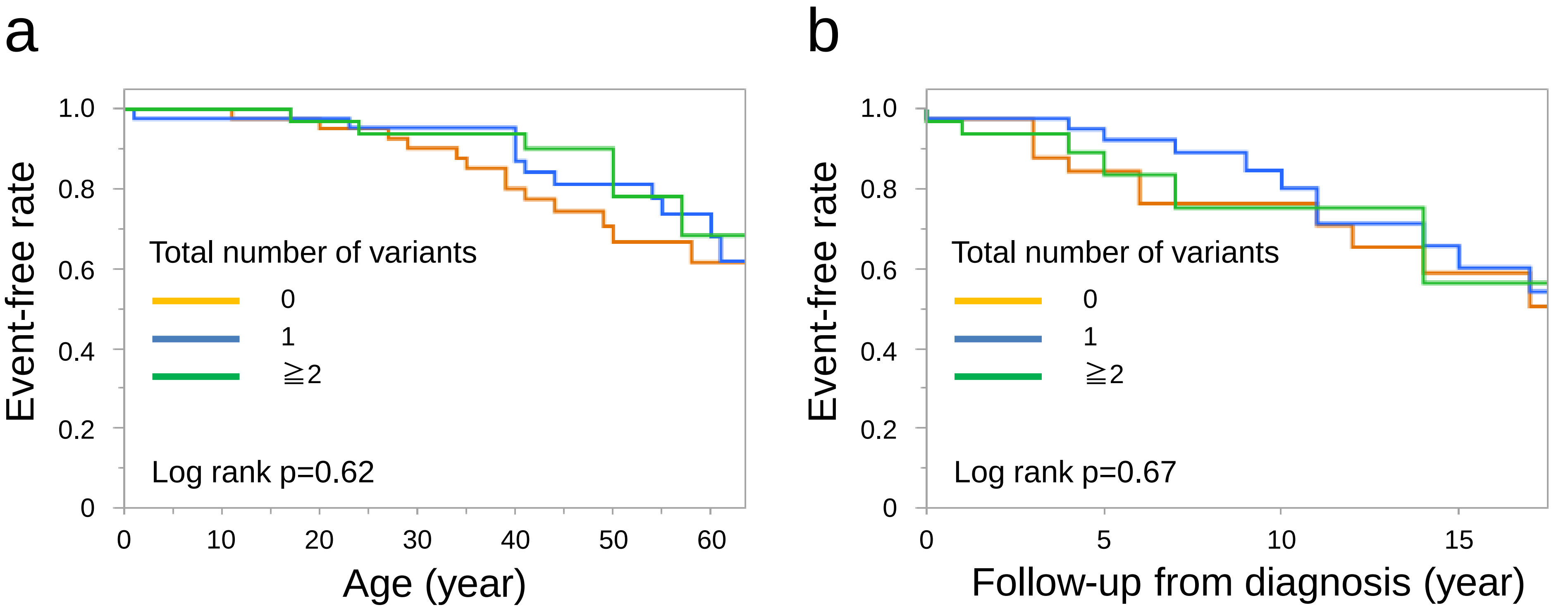

Supplementary Figure Legends

Supplementary Figure 1. Detailed genetic profiles of cardiomyopathies.

Detailed genetic profiles of DCM (a) and HCM (b) are represented. Genes closely linked to cardiomyopathy (yellow), arrhythmias (light green), Noonan syndrome (pink), and Marfan syndrome (light blue) are shown. Colored cells represent the presence of PM (orange), VUS (blue), or PM and VUS (black).

DCM, dilated cardiomyopathy; HCM, hypertrophic cardiomyopathy; PM, pathogenic mutation; VUS, variant of uncertain significance.

Supplementary Figure 2. Associations between clinical outcomes and variant class.

Kaplan–Meier curves illustrating heart transplant- or death-free survival of the patients with PM (n = 37), VUS only (n = 41), and no variants (n = 42) throughout lifespan (a) and during follow-up (b). Probability values were calculated by log-rank tests.

PM, pathogenic mutation; VUS, variant of uncertain significance.

Supplementary Figure 3. Associations between clinical outcomes and total number of variants.

Kaplan–Meier curves illustrating heart transplant- or death-free survival of the patients with no variants (n = 42), one variant (n = 45), and more than one variants (n = 33) throughout lifespan (a) and during follow-up (b). Probability values were calculated by log-rank tests.
